# Mucinase from *Bacteroidesthetaiotaomicron* as mucolytic for mucinous cancer pseudomyxoma peritonei

**DOI:** 10.3389/fmicb.2026.1722421

**Published:** 2026-05-01

**Authors:** Jun Liu, Lingpeng Zhan, Chengjing Xu, Runling Wei, Chengyu Bai, Miao Guan, Zhenglong Sun

**Affiliations:** 1 Interdisciplinary Research Group on Microscopic Imaging and Drug Development, Shenzhen Bay Laboratory, Shenzhen, China; 2 Institute of Chemical Biology, Shenzhen Bay Laboratory, Shenzhen, China; 3 School of Food Science and Pharmaceutical Engineering, Nanjing Normal University, Nanjing, China; 4 College of Life Science, Northwest A&F University, Xianyang, China; 5 School of Life Science and Biopharmaceutics, Shenyang Pharmaceutical University, Shenyang, China

**Keywords:** *Bacteroides thetaiotaomicron*, M60 metalloproteinase, mucinases, mucinous carcinoma, mucolytic therapy, pseudomyxoma peritonei

## Abstract

Mucinous cancers are characterized by tumor gland cells secreting copious amounts of mucus that forms gel-like structures. Pseudomyxoma peritonei (PMP), a rare yet prototypical mucinous malignancy, exemplifies this pathology. Biologically inert mucus coats tumor cells, promoting colonization, immune evasion, and chemoresistance. Pathological mucus accumulation leads to intra-abdominal hypertension, refractory abdominal pain, abdominal adhesions, and intestinal obstruction because of the lack of rapid, efficient, and safe clinical strategies for mucin clearance. Current management relies on extensive cytoreductive surgery to excise tumor masses and adherent mucus. Here, we present an integrated workflow spanning intestinal probiotic protease discovery for the development of mucolytic therapy. By cultivating *Bacteroides thetaiotaomicron* (*B. theta*) with PMP-derived mucus as the sole nutrient source, we identified its intrinsic mucolytic capacity. Crucially, transcriptomic analysis revealed that *B. theta* orchestrates mucus degradation via the PULs gene cluster, with concomitant upregulation of the M60 family metalloproteinases BT4244, BT3015, and BT4272. Functional validation classified these enzymes as “mucinases” responsible for tumor-associated mucin cleavage and mucolysis. Our study provides a foundation for enzyme-targeted PMP therapy, offering a paradigm shift toward minimally invasive, high-efficacy mucolysis. Our findings highlight the translational potential of microbial proteases in addressing clinical challenges posed by mucinous malignancies.

## Introduction

Mucus is composed of heavily glycosylated MUC family proteins interconnected via disulfide and isopeptide bonds, supplemented by trace amounts of cytokines, polysaccharides, and nucleic acids (Recktenwald and Hansson, 2016; Recktenwald et al., 2017). These components collectively form a viscoelastic gel stabilized by reversible physical interactions, including hydrophobic forces and polymer chain entanglements, and reinforced by electrostatic repulsion between negatively charged polysaccharide side chains (Schipper et al., 2007; Jin et al., 2017; Taylor Nordgård and Draget, 2015). The gelling properties of mucus are primarily attributed to the molecular network barrier formed by mucins (Netsomboon and Bernkop-Schnürch, 2016; Georgiades et al., 2014). Notably, serine/threonine tandem repeat domains serve as scaffolds for O-glycosylation, enabling a single mucin molecule to display hundreds of glycan motifs that establish localized glycan density gradients (McShane et al., 2021; Tabatabaei et al., 2015). Extensive glycosylation endows mucins with exceptional hydration capacity and proteolytic resistance, which are critical for maintaining mucosal barrier integrity. Physiologically, this mucus layer lubricates the epithelial surfaces of ocular, oral, and colonic epithelia and prevents direct luminal contact with underlying tissues (Taylor Nordgård and Draget, 2015; Argüeso and Gipson, 2001; Kho, 2018; Tabak, 1995; Celli et al., 2007). However, dysregulated mucin secretion occurs in pathological conditions such as chronic obstructive pulmonary disease (COPD) and mucinous carcinomas (Kufe, 2009; Wedzicha, 2017; Carr et al., 2016). The clinical management of these diseases presents substantial therapeutic challenges.

PMP, a prototypical mucinous malignancy, arises from appendiceal neoplasms with rare colorectal or ovarian origins (Liyan et al., 2015). This disease is characterized by peritoneal dissemination of mucinous implants and ascites, causing visceral compression and organ dysfunction. The current standard of care involves cytoreductive surgery (CRS) combined with hyperthermic intraperitoneal chemotherapy (HIPEC) (Carr et al., 2016). Nevertheless, PMP tumor cells encapsulated within mucin sheaths exhibit free intraperitoneal mobility, facilitating widespread metastasis to sites such as the lesser omentum, hepatic surfaces, and hilar regions (Meyers, 1973; Rizvi et al., 2018; Mittal et al., 2017). Such advanced dissemination often renders CRS ineffective, compounded by the role of the mucin barrier in immune evasion and chemotherapeutic resistance (Bafna et al., 2009; Karlsson et al., 1993; Lechanteur et al., 2018). Furthermore, contraindications to HIPEC and incomplete cytoreduction limit therapeutic outcomes, underscoring the urgent need for novel mucolytic strategies to enable minimally invasive intervention (Ceelen et al., 2021; Lambert, 2015; Guo et al., 2011; Govaerts et al., 2020). While enzymatic mucolysis represents a promising therapeutic strategy, existing approaches show limited efficacy against rigid mucus matrices and necessitate invasive procedures, with potential toxicity risks. These limitations highlight the demand for highly specific, low-toxicity mucinases (Pedram et al., 2023; Beatson and Burchell, 2023; Pillai et al., 2014; Valle et al., 2021; Pillai et al., 2017).

Mucus degradation primarily involves disrupting the mucin-based molecular network (Carlson et al., 2018), relying on three interdependent mechanisms: (1) the enzymatic cleavage of mucin glycoproteins by proteases and glycosidases (Grys et al., 2005; Katoh et al., 2023; Martens et al., 2008; Gutiérrez-Jiménez et al., 2008); (2) the reduction of disulfide bonds via enzymatic or chemical reductants (Addante et al., 2023); and (3) the modulation of mucin polymer rheology through physicochemical interactions with compounds such as glucans or glyceraldehyde ethers (Ishibashi et al., 2010).

Critically, safety considerations are paramount when developing mucolytic agents for *in vivo* applications, necessitating the identification of enzymes with optimal biocompatibility and therapeutic efficacy.

*B. theta*, a well-characterized probiotic symbiont, emerges as a compelling candidate. *B. theta* exhibits exceptional mucolytic activity while maintaining a favorable safety profile because of its intrinsic role in intestinal homeostasis (Luis et al., 2021; Porter et al., 2018). Through its degradation of complex carbohydrates and subsequent production of short-chain fatty acids (SCFAs), *B. theta* enhances gut microbiota diversity, supports cardiovascular health, and promotes mucosal barrier integrity. Notably, its substrate-specific transcriptional regulation enables precise expression of specialized enzymes for degrading diverse substrates, including N-glycans, sulfated polysaccharides, pectins, and O-glycans (Gutiérrez-Jiménez et al., 2008; Luis et al., 2021; Briliūtė et al., 2019; Li et al., 2021). These attributes suggest that *B. theta* represents a tractable and safe platform for the production of mucin-targeting enzymes.

In this study, we cultured *B. theta* with PMP-derived mucus as the sole carbon source, revealing its ability to metabolize tumor-associated mucins. RNA-seq analysis revealed upregulation of the PULs gene cluster and M60 family metalloproteinases (BT3015, BT4244, BT4272). Functional characterization confirmed that these enzymes are dual-specificity mucinases that require both O-glycan recognition, particularly sialic acid modifications, and peptide backbone cleavage at serine/threonine glycosylation sites. Crucially, these mucinases effectively degraded PMP mucus *ex vivo*, supporting their therapeutic potential for targeted mucolysis. Our findings establish *B. theta-*derived M60 metalloproteinases as novel candidates for PMP therapy. By simultaneously targeting glycan and peptide moieties, these enzymes may enable minimally invasive mucin clearance, thereby enhancing CRS/HIPEC efficacy and patient outcomes.

## Methods and data analysis

### *B. theta* culture for transcriptome analysis, growth curve measurement, and imaging analysis

*B. theta* ATCC 29148 was purchased from BeNa Culture Collection (Beijing, CN) in a lyophilized form. Lyophilized bacteria dissolved in PBS were transferred to CNA plates for recovery and anaerobic culture at 37 °C for 3–4 days. Afterward, colonies were selected for expansion culture in FT medium, and the bacteria were collected by centrifugation and supplemented with 30% glycerin for preservation at −80 °C until use.

The FT culture medium powder was dissolved in boiling water according to the instructions and then promptly transferred to an 18 mL universal anaerobic tube (R, reference). The tube was sealed with a butyl rubber stopper and threaded cap. Sterilization was performed at 121 °C for 15 min. For the experimental group, the sterilized and cooled FT medium (R) was slowly supplemented with an additional 0.5% (w/v) PMP-derived purified mucin (RWM) and gently shaken to prevent clumping.

In both Groups R and RWM, the same volume of bacterial liquid suspended in PBS (1 mL, 1*10^7^ cfu/mL) was inoculated into separate anaerobic tubes, which were then incubated at 37 °C, inverting every 2 h. The OD600 was measured at 0 h, 3 h, 6 h, 10 h, 22 h, 29 h, 35 h, and 45 h using a SpectraMax M device (Molecular Devices, Shanghai). The baseline for data deduction in each group is represented by the OD600 measurement taken at 0 h. Each sample was measured three times.

At 29 h, the syringe was inserted into the butyl plug of the anaerobic tube, and 200 μL of medium from each of the two culture groups was added, followed by 600 μL of PBS. The mixture was gently mixed and then centrifuged at 2,000*g for 30 s. Next, 2 μL of the mixed solution was taken to prepare slides using the smear method to observe the morphology. In addition, the slides were squashed using a mixed solution that still contained lumps of mucus.

### Mucinase expression and purification

The ORFs encoding three M60 mucinases were amplified from *B. theta* genomic DNA using primer pairs (BT4244 F/R, BT3015 F/R, and BT4272 F/R). The signal peptide sequence, predicted using SignalP 5.0 tools, was replaced with an 8 × His tag using the same primers.

The expression plasmid pBAD was linearized with a primer pair (pBAD F/R), and a homologous arm structure complementary to the above protein-encoding nucleotide sequence was introduced into the nucleic acid chain 5′ by this primer. The pBAD-BT3015, pBAD-BT4244, and pBAD-BT4272 expression vectors were constructed using homologous recombinant Hieff Clone (Yeasen #10923ES). The plasmid was subsequently transformed into top 10 strains of *E. coli* for protein expression and purification (see Supplementary Figure S2). Cells were cultured in LB medium until OD600 reached 0.5, induced with 0.05% arabinose for an additional 3 h, and disrupted using a pressurized homogenizer (Avestin, Canada) in lysis buffer containing 50 mM Tris–HCl (pH 7.5) and 50 mM NaCl. The resulting lysate, containing the target protein, was purified to >90% purity using sequential Ni-NTA and Superdex-200 pg. 16/600 column chromatography. Finally, glycerol was added to a final concentration of 25%, and the protein was stored at −80 °C.

### Protein digestion

Commercially available glycosylated proteins, including the non-mucin-like Fetuin-A (Sino-Biological #10318-H08H) with O-glycosylation, mucin domain-containing PSGL-1 (Sino-Biological #13863-H08H), MUC-16 (UABio #UA010258), MUC1 (absin:#abs06227), PODXL2 (MCE #HY-P78020), C1-INH (R&D #2488-PI-200), N-glycosylated CD47 (absin #abs05025), and BSA, along with the prokaryotic cell protein SpNanA, which is non-glycosylated, were utilized as substrates for protease digestion. To evaluate the activity of the three mucinases against mucin-like glycoproteins and non-mucins, the reaction conditions were as follows: a 1:4 E/S ratio, a total volume of 10 μL, and in PBS. A portion of each sample (0.5 μg) was loaded onto a 10% Tris–Gly SDS-PAGE gel and run at 180 V for 1 h. Each gel was stained with Coomassie G-250 Ultrafast Staining Solution (Sangon Biotech) according to the manufacturer’s instructions.

The digestion pattern of C1-INH was investigated using EDTA and the removal of glycoprotein sialic acid by neuraminidase. The final EDTA concentration was 10 mM. For reactions requiring the addition of neuraminidase, 10 mU of neuraminidase was added to each reaction. The system was constructed in 10 μL of glyco-buffer supplemented with 2 μL of pH 6.5 and 200 mM Tris–HCl at 37 °C for 3 h.

After simple washing and dialysis homogenization of the mucus from volunteer PMP patients, the moisture content was measured by drying to facilitate subsequent experiments in which the PMP mucus could be directly weighed in its wet state. To assess the digestion activity of the three enzymes, the PMP-derived mucus was supplemented with Mucinase at a ratio of 300 μg of mucin per 30 mg of mucus (by dry weight) and continuously incubated at 37 °C until use. Changes in the interior of the mucus gel were observed after centrifugation at 15,000 g for 5 min. The same batch of samples was reswirled. By adjusting the height of the upper cone plate to 5 mm, all the reacted mucus was loaded into the pores of the cone plate, and the shear viscosity was analyzed at a frequency of 10/s in the upper cone plate. The experiment was conducted using three replicates per group, yielding 12 viscosity measurements per sample within a 1-min time frame.

### Mass spectrometry sample preparation

C1INH samples (2 μg) were dissolved in 5 mM CaCl_2_ and 50 mM sodium acetate (pH 5.5) and incubated for 3 h with 100 mU sialidase NA (neuraminidase; Sangon Biotech #A004759) when desialylation was needed. Afterward, 0.3 μg of each M60 metalloproteinase was added to a 25 μL system for overnight digestion, and the solution contained a 50% ratio of isotopic water H_2_^18^O. At the end of the incubation, the solution was dried at 25 °C using a SpeedVac device. A heavy suspension of 50 μL of 50 mM ammonium bicarbonate solution was added. Next, 3 μL of 50 mM DTT was added for reduction, and the solution was incubated for 1 h at 60 °C. After that, 2 μL of 100 mM IAA was immediately added to the solution for alkylation, and the mixture was incubated for 30 min at room temperature in the dark. At the end of the incubation period, 50 μL of 50 mM ammonium bicarbonate buffer was added to dilute the unreacted IAA and other substances. Next, 0.5 μg of mass spectrometry-grade trypsin (Promega, # V5280) was added, and the samples were incubated overnight at 37 °C. The reaction was terminated by adding 1 μL of formic acid. Peptides were extracted by SPE using a monospin C18 resin (GL Sciences). In brief, after washing the methanol-activated fillers once with 80% acetonitrile, the fillers were washed twice with ultrapure water containing 0.1% formic acid. The digested peptides were loaded onto the column for binding and were subsequently washed five times using ddH_2_O supplemented with 0.1% formic acid. Finally, the eluate was combined using 80% acetonitrile and acetonitrile. It was concentrated and dried using a SpeedVac device and then dissolved in ultrapure water containing 0.1% formic acid. The concentration was measured using a NanoDrop instrument at 205 nm and adjusted to 250 ng/μl.

### Mass spectrometry data acquisition

The mass spectrometer scanner mode was set as described by Mahoney et al. (2024), with 4 μL loaded per injection. An aqueous solution containing 0.1% formic acid (solvent A) served as the mobile phase. Next, the peptides were incubated on a 15 cm RP-HPLC analytical column (75 μm × 25 cm) fitted with a 3 μm C18 filler (Thermo Fisher, #164569) through three gradient periods: 80% acetonitrile containing 0.1% formic acid over 100 min, a 5–35% gradient of solvent B (80% acetonitrile containing 0.1% formic acid) over 0–75 min, and a 35–50% solvent B concentration change for 75–83 min, followed by a rapid solvent B gradient change of 50–100% for 83–91 min until 100 min.

### Mass spectrometry data analysis

PEAKS mass spectrometry software was used to identify sugars in the raw data according to C1INH protein fasta files. The mass tolerance was set to 10.00 ppm for the MS1 spectrum, 0.02 DA for the MS2 spectrum, and 20.00 ppm for the sugar fragments.

Met oxidation was set as a variable modification, and carbamidomethyl cysteine was set as a fixed modification. The ^18^O labeling of the C-terminus and ^18^O labeling at both C-terminus oxygens were set as variable modifications. Enzymatic hydrolysis was performed in two ways: In the pre-trypsin type, the trypsin enzyme cleaves the C-terminus of R and K of the protein. With reference to the known cleavage peptide of BT4244, the M60 protease cleavage type was generated. That is, files were searched for cleavage at the N-terminus to serine and threonine. Both enzyme processing search modes retain redundancy, with the digest mode set to semispecific and six allowed missed cleavages, with a peptide length of 3–45 aa. The maximum variable number of modifications per peptide segment is 5, the addition product is NH4, and the maximum number is 2. The maximum number of fucose modifications accepted is two molecules. The glycosylation search type was O-linked, and the sugar bank contained 236 sugar types. The data were filtered such that the O-linked peptide score was ≥20, the O-linked glycan score was ≥20, and the protein targeting confidence was −10lgP ≥ 15. In accordance with the method described by Mahoney et al., Byonic software was used to mutually verify the search results of PEAKS online.

### Cell line culture and cytological experiments

Experiments were performed using a panel of cell lines, including normal epithelial cells (HK2 and BEAS-2B), tumor cell lines (HT29, LS174, SKOV-3, and A549), and genetically engineered HEK-293T cells, which were cultured with 5% CO_2_ at 37 °C. In addition to SKOV-3 and HT29 cells, which were cultured in McCoy’s 5A medium (GIBCO cat # 16600082) supplemented with 10% fetal bovine serum and 1% penicillin–streptomycin (GIBCO cat #15140122), the other cells were cultured in basic DMEM/F2 medium (GIBCO cat #11320033) supplemented with 10% fetal bovine serum and 1% penicillin–streptomycin solution. Cells were grown in 10 cm dishes until confluence, digested with 0.15% trypsin for 5–10 min, and collected; cell density was then measured. Each cell type was seeded into 96-well plates at a density of 1,000 cells per well to allow for adherence and acclimation. After 12 h, the medium containing serum and antibiotics was removed, and media containing different concentrations of the three mucinases were added to continue the culture.

Three different mucinases, BT3015, BT4244, and BT4272, were each prepared at an initial concentration of 10 μM in DMEM and McCoy’s 5A medium without serum or antibiotics and then diluted in a series of five-fold dilutions. Once the test cells had adhered and the original medium was removed, the medium was quickly replaced with medium containing mucinases, gently mixed by shaking, and then cultured.

Cell viability was measured using PrestoBlue HS (Invitrogen cat: #A13261) according to the manufacturer’s instructions 24 h after mucinase treatment. Each data point represents the mean ± SD of four wells grown in a 96-well plate, and the data for each treatment were fitted using the Gompertz growth model. For the carcinoma series, data were collected from five wells for this type of cell variation. In the logarithmic coordinate system, the coordinate value for the control group without mucinase was set to 0.1 nM.

### Statistical analysis

Applicable statistical methods were adopted for different data types and described in the figure notes. The data in this study were analyzed using OriginLab software, and the results are presented as the mean ± SD, unless otherwise noted. Unlisted analyses are described in the Supplementary documents.

## Results

### PMP-derived mucus enhances *B. theta* proliferation and induces morphological adaptation

**Figure 1:**
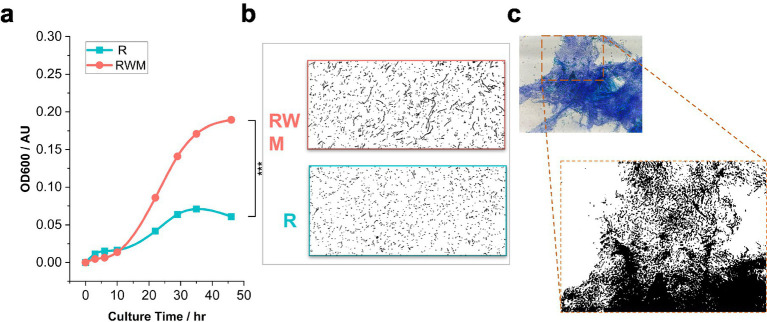
PMP-derived mucus modulates *B. theta* proliferation and morphology. **(a)** Growth kinetics of *B. theta* in control (R) versus PMP mucus-supplemented (RWM) media. Optical density at 600 nm was monitored at the indicated time points (mean ± SEM, *n* = 3 biological replicates). Note that 27 h here refers to the midpoint of the logarithmic phase obtained by fitting manually measured growth curves. Adjusting the goodness of fit ensures that *R*^2^ > 0.98 in the logarithmic phase intervals while considering the overall growth trend over the entire duration. **(b)** Micrographs for grayscale processing (40× objective, NA 0.65) of *B. theta* at 29 h postinoculation, which corresponds to the rectangle in **(a)**. RWM-cultured cells exhibited elongated morphology (2.35 ± 1.18 μm vs. 3.13 ± 2.85 μm in the R group) and chain-like aggregation (scale bars: 50 μm). **(c)** Methylene blue-stained mucin and *B. theta* in RWM cultures. Distinct staining abilities for glycosylated mucin and bacterial surface proteins were observed using methylene blue staining, allowing differentiation between the non-mucus portion, the mucus matrix, and the bacterial cell body. The enlarged image was processed using ImageJ software to display it in grayscale and enhance contrast for better visualization of the bacterial distribution.

Growth curve analysis revealed distinct kinetic profiles between the R (control) and RWM (0.5% PMP mucus-supplemented) groups. Both groups entered the mid-exponential phase at 27 h; however, the RWM group showed prolonged exponential growth characteristics compared to the R group (Figure 1a), with the OD600 at 27 h being significantly greater (0.189 ± 0.060 vs. 0.061 ± 0.004). Microscopic observations conducted at 29 h during sampling further confirmed this difference, showing that the bacterial density in the RWM group remained higher than that in the R group, and the morphology maintained characteristics typical of the exponential phase. Morphological alterations were assessed via methylene blue staining coupled with bright-field microscopy (40× objective). *B. theta* in the RWM group displayed a marked difference in cellular length, measuring 2.35 ± 1.18 μm compared to 3.13 ± 2.85 μm in the R group, even with elongated linear or bead-chain configurations characterized by denser cytoplasmic staining (Figure 1b; Supplementary Figure S1). Notably, RWM-cultured cells formed mucin-adherent aggregates (Figure 1c), suggesting active remodeling of the mucus matrix. While the precise regulatory mechanisms underlying these morphological shifts remain to be elucidated, our findings collectively demonstrate the capacity of *B. theta* to metabolize PMP-derived mucus, which is associated with enhanced proliferation and structural adaptation.

### Transcriptome analysis reveals a mucolysis pattern in *B. theta* synthesis and metabolism

**Figure 2:**
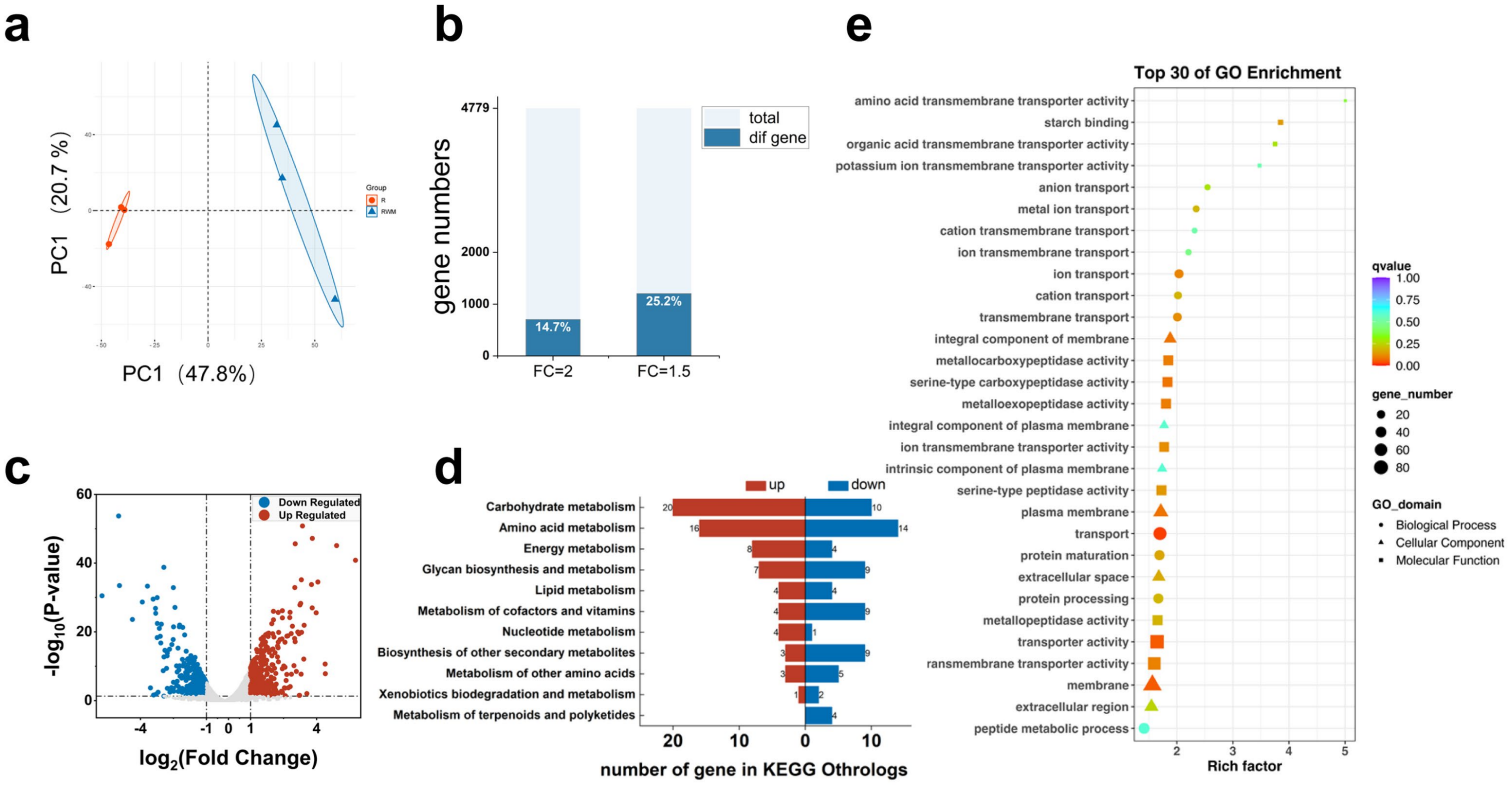
The transcriptome of *B. theta* shows a transcriptional pattern consistent with that associated with mucus supplementation. **(a)** The R and RWM groups were analyzed by PCA based on gene expression. Orange represents the R group, and blue represents the RWM group. The samples deviate further between the groups because of the addition of mucus. The figure was generated by bioinformatics (https://www.bioinformatics.com.cn, last accessed on 10 November 2023), an online platform for data analysis and visualization. **(b)** When the fold change is 1.5 or 2, the ratio of differentially expressed genes to the total genome is controlled. **(c)** Volcano map of gene expression, showing genes whose expression differed between the RWM and R groups at FC = 2 and FDR > 0.01. **(d)** Metabolic pathway data obtained by directly enriching genes whose expression was upregulated or downregulated by KEGG showed the most active changes in genes associated with carbohydrate metabolism and amino acid metabolism. **(e)** The top 30 orthologs for GO term annotations of differentially expressed genes. The size of the shape indicates the number of enriched genes, and the color indicates confidence; the smaller the *q* value, the higher the confidence.

We collected bacteria from cultures of the RWM and R groups at the mid-log growth phase and extracted total RNA for sequencing. Differential gene expression analysis was performed to determine how the bacteria responded to the addition of PMP mucus. After PCA, the clusters significantly differed between the samples processed in the groups, and the greater distance of the samples in the RWM may be due to the complexity of the mucus composition (Figure 2a). Using a lenient FC (FC = 1.5) resulted in a large number of differentially expressed genes (25.2%, *n* = 4,779), and although this FC is beneficial for showcasing the mucus metabolism capabilities of *B. theta*, our goal is to screen for genes closely associated with mucus supplementation. Therefore, we used a more stringent FC value (FC = 2), which still included approximately 14.7% of the 4,779 genes; however, some genes previously reported to be differentially expressed in mucus metabolism were not present in the dataset, as described later (Figure 2b). To gain insight into the types of these differential genes, we aligned them to the KEGG database under the more stringent condition of FC = 2. A comparison of RWM and R revealed that 317 genes were upregulated and 390 genes were downregulated in RWM (Figure 2c). The orthologs of the differentially expressed genes indicate their participation in carbohydrate metabolism and amino acid metabolism, followed by carbohydrate synthesis and metabolic pathways. The KEGG results also revealed 20 and 16 upregulated genes related to carbohydrate metabolism and amino acid metabolism, respectively, which represented the most typical changes in global metabolism. Genes related to glycan synthesis and secondary metabolic synthesis were significantly downregulated. In addition, since many genes involved in lipid anabolism are also related to carbohydrate metabolism, the genes associated with the synthesis of polyketide substances were suppressed and downregulated (Figure 2d).

The bioprocesses in *B. theta* were associated with changes in carbohydrate and energy levels and with the downregulation of cofactors, vitamins, and other secondary metabolites, revealing a pattern in which bacteria rapidly metabolize nutrients from mucus in the RWM.

The differential orthologs enriched in the GO term annotations in the top 30 included 8 membrane transport-related terms and enzyme activity-related terms, including metallocarboxypeptidase, metalloexopeptidase, metallopeptidase, and serine-type peptidase activity (Figure 2e). The starch binding term is ranked second and has been suggested to be involved in the cellular transport and metabolism of amyloid polysaccharides.

### Putative M60-like family proteases are strongly involved in mucus utilization patterns

While metabolic pathway analysis revealed potential mucin degradation capacity, our primary goal was to identify specific mucin-degrading enzymes in *B. theta* that demonstrate substrate specificity.

The *B. theta* genome contains substrate-specific PULs, with all 88 previously characterized (Martens et al., 2008) PULs being functionally categorized into O-glycan processors, N-glycan processors, host metabolism-associated clusters, and uncharacterized (UK) classes lacking experimentally validated substrates.

**Figure 3:**
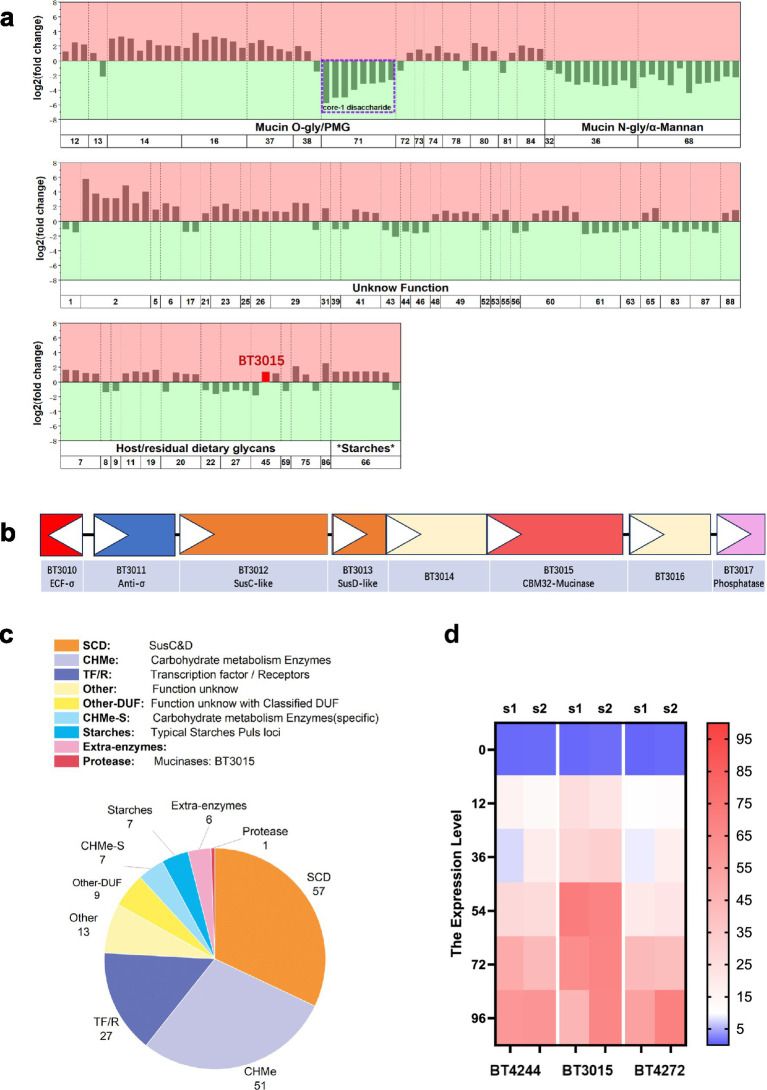
Enzymes involved in the response to mucus degradation activity are part of the *B. theta* O-glycan degradation pathway, including the putative mucinase BT3015. **(a)** The differentially expressed PUL genes associated with PMP mucus addition were enriched in five previously reported types of PULs and are shown in the chart. The upregulated genes are displayed in red, while the downregulated genes are displayed in green. PUL genes of the mucin O-gly type associated with core-1 disaccharides are highlighted in purple boxes. PUL-45 is shown in the host/residual dietary glycan type; the position of BT3015 is highlighted in red. **(c)** A typical PUL gene cluster with *σ* factor, a dissociable subunit of RNA polymerase, as the regulatory element, except BT3015, which has a degradable mucin polypeptide backbone and contains SusC (an outer membrane TonB-dependent transporter) and SusD (a surface glycan-binding protein) as well as phosphatases that may be involved in polysaccharide degradation. White triangles indicate the direction of transcription. **(b)** The functional classification of the differentially expressed genes enriched in PULs showed the function and number of different proteins in *B. theta* using PMP mucus. **(d)** RNA transcription analysis of culture maps with extended incubation times using qRT–PCR. Each data point represents the expression level of two individual biological samples, S1 to S2, but there are no technical replicates. The results revealed the expression regulation of the underlying time progression.

The functional classification of differentially expressed genes within PULs revealed substantial enrichment of SusC/D-type membrane receptors and proteins associated with mucin degradation pathways. Overall, PULs involved in mucin O-glycan processing were upregulated, whereas those associated with N-glycan and α-mannan metabolism were predominantly downregulated. Notably, in the canonical single-starch utilization system, the Sus PUL genes in *B. theta* also responded to PMP mucus supplementation (Figure 3a). The observed upregulation of O-glycan PUL clusters correlated with the extensive O-glycosylation pattern of intestinal mucus. In PUL-45, the *σ* factor BT3010 was downregulated, whereas the mucolytic enzymes BT3015, BT3013, and BT3017 were upregulated. The characteristic sus paradigm and σ factor PULs are illustrated as examples with the BT3015 gene cluster (Figure 3b). However, expression data for the phosphatase BT3017 were excluded from Figure 3 because of its absence in control group transcriptomes, precluding fold-change calculations. A similar situation occurred with the galactose-binding-like protein BT0266 in PUL-5. Crucially, differential expression analysis revealed significant upregulation of BT3015 (Figure 3c), a putative protease containing both F5/8 Type C and peptidase M60-like domains, suggesting M60 family metalloprotease functionality. BT3015 resides within an O-glycan-processing PUL regulated by an ECF-*σ*/anti-σ system specialized for host-derived substrates—a system previously shown to control the degradation of breast milk oligosaccharides and intestinal mucin O-glycans (Martens et al., 2008).

Previous studies have reported that the mucinase properties of BT4244 are upregulated in mucus-fed conditions, and recent studies have reported that three proteases, BT4244, BT3015, and BT4272, belong to the same metallopeptidase family in *B. theta* and can degrade BSM, but the degradation activity of these proteases on tumor mucins has not been confirmed (Nakjang et al., 2012; Trastoy et al., 2020; Shon et al., 2020; Ndeh, 2013; Ndeh et al., 2025; Kostopoulos et al., 2021).

Moreover, a literature review revealed that a genetic locus is involved in mucin O-glycoprotein processing and N-acetylgalactosamine metabolism; thus, we further validated the expression characteristics of the PUL-78 locus in early culture and later stages. BT4240-BT4243 in PUL-78 was upregulated in the experimental group (RWM-27), which was significantly different from that in the control group (R), but the expression of BT4244 was not significantly different at 27 h. However, the time-delayed results for *B. theta* revealed that expression of both the PUL-78 and BT4272 genes was significantly upregulated in RWM-71 cultures compared with that in RWM-27 cultures. Specifically, upon the addition of mucus to the basal medium, BT3015 was expressed first at approximately 27 h according to the RNA-seq data, followed by the expression of the BT4244 and BT4272 genes at a later stage (see Supplementary Figure S6).

Moreover, BT3015 was found only in the upregulated expression transcriptome sequencing results, while BT4244 and BT4272 were not observed in the differential expression gene list; thus, we investigated the cause and validated gene expression by introducing time-delayed culture conditions for BT4244, BT3015, and BT4272. BT3015 achieved high expression earlier than the other two proteins in the RWM (Figure 3d).

Previously reported studies and our own validation experiments confirm that the metalloproteases BT3015, BT4244, and BT4272 of *B. theta* are related to mucin degradation, have intrinsic regulatory expression mechanisms, and are expressed at different stages of cell growth.

### The putative M60 family proteins BT3015 and BT4272 are identified as mucinases in accordance with BT4244

**Figure 4:**
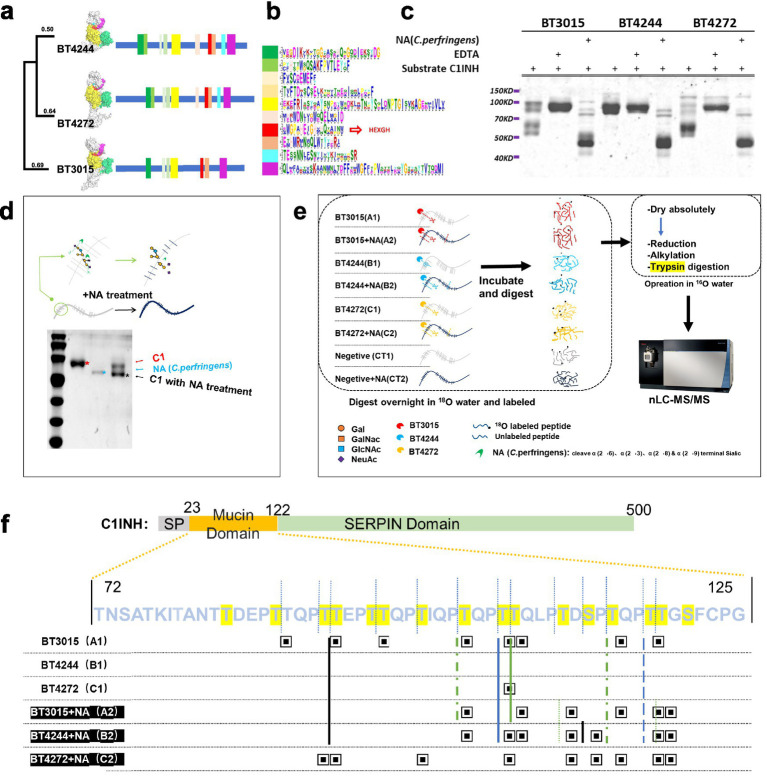
By comparing BT3015 and BT4272 with BT4244, structural and activity analyses revealed that both were mucinases. **(a)** Evolutionary distances of the three M60 metalloproteinases, BT3015, BT4244, and BT4272. Domains with potential relationships are visualized in color with PyMOL. Cyan: potential carbohydrate binding domains with unknown functions. Red: carbohydrate binding domains of the CBM32 family. Orange: the overall domain containing the catalytic domain, for which a crystal structure has been obtained for BT4244 and BT3015. The numbers at the end of the tree show the evolutionary distances obtained by comparing amino acid sequences between different proteins. **(b)** Multiple sets of sequence-homologous motifs shared by BT3015, BT4244, and BT4272. The top 10 sequences according to confidence priority are presented. **(c)** The digestion product bands of C1INH treated with the three mucinases using 10 mM EDTA or 1 mU of neuraminidase are shown. The arrows indicate the addition of neuraminidase and C1INH. **(d)** NA treatment resulted in a change in the molecular weight of C1INH but did not result in the formation of a degradation band similar to that resulting from mucinase digestion. **(e)** Experimental method design for high-resolution nanoliter liquid-coupled mass spectrometry. **(f)** Comparison of restriction sites obtained from different treatments using mucinase. The structure of the C1INH protein is shown in various colors, the amino acid sequence of its mucin domain is displayed, and the glycosylation site at this location is marked in bright yellow. The same sites between different treatments are connected by lines. SP, signal peptide.

The *B. theta* genome contains only three peptidase M60 domain proteins, BT3015, BT4244, and BT4272, according to searches for primary structural motifs. By constructing evolutionary trees and comparing and analyzing secondary and three-dimensional structures, we can conclude that the structure is more conserved than the sequence; BT3015, BT4244, and BT4272 have similar structures and share only a modest number of common ancestral sequences, having evolved into protein members with distinct functional differences (Figure 4a). Here, we also show the top 10 homologous motifs based on MEME Suite searches, using similar colors corresponding to sequence structure and highlighting in red the catalytically active site, which shares a catalytic domain containing HEXGH motif homology (Figure 4b).

First, we evaluated the putative mucinase activity of multiple protein substrates as described in Table 1 and Supplementary Figure S3. BT3015 and BT4272 are proteases that cleave proteins containing a mucin domain but do not cleave proteins lacking a mucin domain. None of the three enzymes digested N-glycosylated CD47, non-glycosylated BSA, or the prokaryotic cell protein SpNanA. BT4244 showed no cleavage activity toward most proteins, but cleavage activity was observed after coincubation with an additional sialidase, indicating intolerance to sialic acid modifications. Interestingly, while both BT3015 and BT4272 cleaved C1INH, they exhibited distinct cleavage patterns (Supplementary Figure S3; Figure 4c). In summary, BT3015, BT4244, and BT4272 demonstrated specific activity against O-glycosylated proteins containing a mucin domain and met the definition of “mucinases.”

**Table 1:** Cleavage ability of multiple substrate proteins with the *B. theta* M60 family proteins BT3015, BT4244, and BT4272.

Substrate	#Type	BT3015	BT4244	BT4272
Fetuin A	#A	−	−	−
PSGL-1	#B	+	+	+
Muc16	#B	+	−	+
CD47	#C	−	−	−
PODXL2	#B	+	−	−
Muc1	#B	+	−	−
C1-INH	#B	++	−	+
BSA	#N	−	−	−
SpNanA	#N	−	−	−

A. O-glycosylated non-mucin domain glycoproteins.

B. O-glycosylated mucin domain (50% with O-glycosylation) glycoprotein.

C. N-glycosylated protein.

N. Non-glycosylated protein.

“−”: No degradation was observed.

“+”: Minor degradation.

“++”: Apparent degradation.

The structural features of C1INH are well established, with glycosylations accounting for more than 40% of its mass (Figure 4f). It is widely used to characterize mucinase activity. Given this, we selected C1INH as the substrate for subsequent experiments. The results of C1INH after treatment with the three mucinases revealed that BT3015 had the strongest degradation activity, followed by BT4272, whereas BT4244 did not degrade natural C1INH, which was non-desialylated. Furthermore, C1INH showed significant and distinct degradation patterns with the three mucinases. However, consistent digestion bands were observed when the mucinases acted on desialylated C1INH. Finally, the activity of all three mucinases was completely inhibited by 10 mM EDTA (Figure 4).

Removal of terminal sialic acid by *Clostridium perfringens* neuraminidase resulted in a decreased molecular weight of C1INH, as observed via gel electrophoresis (Figure 4d).

We performed mass spectrometry experiments using the outlined procedure to enable isotopic labeling of peptides generated by digesting substrates with mucinases from the three M60 families (Figure 4e). The C-termini of the peptides were modified via hydrolysis with 18O-labeled water, resulting in a +2 Da mass shift, thereby distinguishing them from trypsin-hydrolyzed peptides. Since the mucinase activity of BT4244 is strongly inhibited by sialic acid modifications at the glycopeptide glycan termini, the removal of sialic acid using the sialidase NA was essential.

Glycosylation sites and mucinase cleavage sites within the mucin domain of C1INH were identified by mass spectrometry. Cleavage sites were determined by analyzing discontinuities in characteristic peptide fingerprints (Figure 4e). Common cleavage sites shared across treatment groups (excluding the negative control) were visualized using dot-joining plots to demonstrate site conservation (Figure 4f; Supplementary Figure S4). The raw mass spectrometry data were analyzed and validated using PEAKS and Bionic software. The identified peptides were predominantly concentrated in residues 85–119 of the C1INH mucin domain. Analysis of peptides generated by mucinase-treated C1INH revealed a consensus cleavage motif, X↓(T/S*), where:

- ↓ = cleavage site.
- T/S* = represents O-glycosylated threonine or serine.

All three mucinases exhibited consistent polypeptide sequence specificity at cleavage sites. This mechanistic consistency may explain the similar C1INH degradation band patterns observed for all three enzymes following sialidase NA treatment to remove sialic acid. However, these results do not account for the differences in degradation band patterns between BT3015 and BT4272 observed without sialidase NA treatment. In summary, while the observed regulatory patterns may reflect both protease specificity and broader substrate utilization strategies, these findings strongly support the specialized mucolytic capacity of *B. theta*. We therefore propose the classification of these M60 proteases as bona fide mucinases.

We propose that this divergence arises from distinct recognition specificities toward glycosylated glycans and may be related to different types of sialic acid modifications at specific locations. Additionally, our data confirm that all the serine and threonine residues within this C1INH domain are O-glycosylated—a finding not previously reported.

### M60 mucinase degrades PMP mucus *in vitro*

As shown in Supplementary Figure S5, the PMP mucus derived from the patient appeared as a semisolid gel, indicating abnormal growth in the patient’s body. PMP mucus degraded differently when treated with the three distinct mucinases.

**Figure 5:**
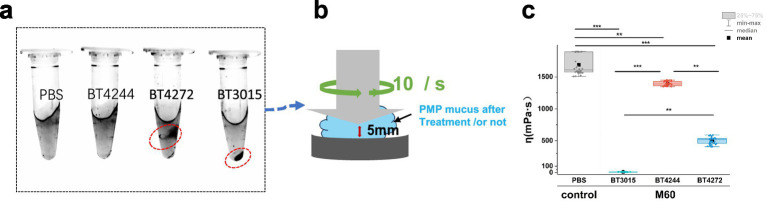
Mucinase from *B. theta* can degrade PMP tumor-derived mucus *in vitro*. **(a)** PMP mucus treated with the three enzymes exhibited different gel states, and the morphological changes were further analyzed using a cone-plate viscometer. Each reaction contained 10.0 ± 0.2 mg dry-weight fully hydrated mucus incubated for 48 h at a 1:100 enzyme-to-substrate ratio, depicting direct observations of mucus degradation. **(b)** Schematic diagram of the viscosity measurement model for mucus treated with three M60 enzymes. **(c)** BT3015 reduced the viscosity to Δη = 7.7 mPa·s, indicating that the mucus was nearly liquefied. BT4272 yielded Δη = 500.6 mPa·s with residual mucous aggregates. BT4244 showed a minimal reduction (Δη = 1394.2 mPa·s). Compared with the control group, all the enzyme-treated groups presented significantly lower viscosity (Δη = 1,691 mPa·s). Differences in viscosity after mucinase treatment between groups were assessed using the Kruskal–Wallis test with Dunn’s *post hoc* test (3 biological replicates per group and 12 rheological measurements per sample). Significance levels: *p < 0.05, **p < 0.001, ***p < 0.0001.

Figure 5a clearly shows that BT3015 completely degraded the gel-like mucus, resulting in the total disappearance of the gel-like polymer. BT4272 exhibited limited mucus degradation with centrifugation, revealing a reduced amount of gel-like mucus. Visual inspection revealed no difference between the BT4244 and control groups. After centrifugation, no heterogeneous precipitate accumulated in the tubes of the BT4244 or control group. However, when the mucus incubated with BT4272 was subjected to high-speed centrifugation, heterogeneous particles clearly formed in the middle of the tube. This phenomenon aligns with the principles of density gradient centrifugation: incomplete liquefaction preserved mucus viscosity, causing degraded precipitates to aggregate mid-tube. In contrast, BT3015 fully liquefied the mucus, allowing insoluble precipitates to settle at the bottom of the tube in minimal volume, confirming the loss of solution viscosity.

To measure viscosity, centrifuged samples exhibiting mucus dissolution were rehomogenized by high-speed vortex mixing and loaded into the rheometer’s cone-plate viscometer. Shear viscosity was measured for 10 mg of degraded mucus using a cone-plate viscometer (Figure 5b). All three mucinases exhibited varying degradation efficacy (Figure 5c); BT3015 demonstrated the highest activity, fully liquefying mucus, followed by BT4272 and BT4244, whose effects were negligible.

### Cytological experiments of M60 mucinase

**Figure 6:**
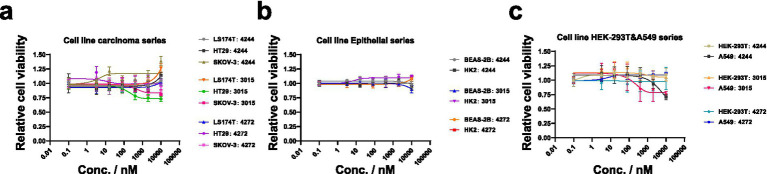
Cell viability of different cell types after treatment with mucinase and its trend with mucinase concentration change: **(a)** the carcinoma series includes SKOV-3, HT29, and LS174T cells. These cells, reported in previous studies to produce mucus, are derived from carcinoma or adenocarcinoma. After treatment with different enzymes, the cell lines showed differential sensitivity to the enzymes; **(b)** the cells in this group were derived from immortalized epithelial cells, including BEAS-2B and HK2 cells. After treatment with 10 μM mucinase, cell viability did not significantly change, cell proliferation was normal, and morphology remained largely unchanged; **(c)** this group lacks clear categorization. For example, A549 cells originate from lung tumor cells but do not have mucus-secreting characteristics; HEK293T cells originate from kidney epithelial cells but have been artificially modified to integrate viral genes. The most important feature of this group is that cell-specific responses are more pronounced. The activity of A549 cells is significantly affected by specific enzymes, whereas the overall activity of HEK293T cells is not affected, but they exhibit spheroid growth.

To confirm the feasibility of this mucus degradation approach, it is necessary to verify that these mucinases are non-toxic proteins that do not negatively affect all cells. We evaluated several cell lines and ultimately confirmed that the effects of these three mucinases depend on their activity and vary across different cells. First, for mucus-producing cells, the broad-spectrum substrate mucinase BT3015 reduced the viability of HT29 and SKOV-3 cells due to its stronger sialic acid tolerance. The other enzymes, which have lower protein cleavage efficiency, did not negatively affect cell growth and, at high concentrations, might even act as protein sources, thereby increasing cell vitality (Figure 6a). In epithelial cell lines, treatment with enzymes at concentrations of at least 10 μM had no significant effect on cell viability (Figure 6b). In the A549 non-small-cell lung cancer cell line, cell viability decreased significantly under treatment with BT4272 and BT3015 (Figure 6c), and the cells did not proliferate. In HEK293T cells, enzyme treatment did not affect cell viability; however, microscopic observations revealed that the cells grew in clusters and formed spheroids (see Figure 6c; Supplementary Figure S7).

## Discussion

Current research on mucinous cancers suggests that the development of mucus-targeting enzymes inspired by bacterial adaptations for mucin degradation represents a promising therapeutic direction. Although bacterial proteases can target human mucins (Pedram et al., 2023; Grys et al., 2006; Hews et al., 2017), such as lectin-like proteases that act on leukocytes and human gut O-glycopeptidase OgpA-processing mucins (Trastoy et al., 2020), their efficiency is limited by substrate specificity and glycosylation patterns (Shon et al., 2020; Chongsaritsinsuk et al., 2023; Haurat et al., 2020; Wardman et al., 2023), resulting in cytotoxicity and hemagglutination (Gutiérrez-Jiménez et al., 2008; Ayala-Lujan et al., 2014). While murine models indicate risks of brain hemorrhage and inflammatory responses, a recent study using a modified recombinant mucinase from pathogenic bacteria showed very promising results *in vitro* and *in vivo* for the treatment of mucinous tumors. However, mouse models indicate a risk of cerebral hemorrhage and an acute inflammatory response (Pedram et al., 2023). These characteristics limit their use in the treatment of mucinous cancers; thus, further optimization is needed.

*Bacteroides thetaiotaomicron* (*B. theta*), a beneficial gut commensal, efficiently degrades intestinal mucins by precisely adapting to mucosal heterogeneity. To exploit this capability, we harvested clinical PMP mucus to identify bacterial proteases that degrade pathological mucins. The results confirmed the bacterial degradation of PMP-derived mucus. Notably, mucins exhibit intrinsic resistance to bacterial degradation through three mechanisms: (1) highly repetitive, glycosylated PTS (tandem repeat) domains and a crosslinked cysD domain (Jiang et al., 2021; Sheng and Hasnain, 2022; Paone and Cani, 2020; Lang et al., 2016); (2) a PTS domain that provides variable, densely glycosylated side chains (Jiang et al., 2021; Taleb et al., 2022; Carlstedt and Sheehan, 1989; Gallego et al., 2023); and (3) terminal sialic acid/sulfate modifications that create negatively charged barriers hindering enzymatic access to peptide backbones (Katoh et al., 2023; Gallego et al., 2023; Schauer, 2009; Luo et al., 2014; Yao et al., 2022). Despite these barriers, *B. theta* exhibits exceptional mucin utilization efficiency. To elucidate its degradation mechanism for highly glycosylated proteins, we performed RNA-seq analysis of *B. theta* grown on PMP mucus to assess global transcriptomic responses to mucin metabolism.

Consistent with Athmaj’s findings on the nutritional dependencies of *B. theta*, our RWM group observed deeper bacterial cell surface staining and similar morphology, suggesting abundant expression of membrane-bound SusC and SusD proteins (Rangarajan et al., 2020). We propose that mucus degradation occurs primarily extracellularly, with degradation products subsequently transported intracellularly, a process requiring high concentrations of membrane proteins. In Bacteroides, substrate-degrading enzymes and transporters are colocalized at polysaccharide utilization loci (PULs) and are regulated by specific factors. Our transcriptomic analysis revealed that genes induced by O-glycosylated PMP mucus (a hallmark of this pathology) cluster within O-glycan-targeting PULs. While most O-glycan-related PULs were upregulated, PUL-71 and the BT4244-BT4250 locus within PUL-78 showed paradoxical downregulation. Martens et al. reported that PUL-71 degrades and transports core-1 disaccharides, which are fundamental units of mucin O-glycosylation present in PMP mucus (Thomsson et al., 2012). The BT4244-BT4250 system is specifically responsive to core-1 disaccharides, which exhibit delayed upregulation during late-phase growth with type III PGM polysaccharide (Martens et al., 2008). We hypothesize that during the logarithmic phase, the complex glycosylation of PMP mucus prevents *B. theta* from releasing core-1 disaccharides, delaying activation of the degradation pathway. This finding is supported by the late-stage upregulation of PUL-78 (BT4244-BT4250) in extended cultures, which aligns with the core-1 disaccharide exposure mechanism.

Based on the metabolic characteristics demonstrated in the results with PMP mucus addition, we are interested in BT3015, BT4244, and BT4272, which are uncharacterized genes with differential gene expression. All three genes were classified as peptidase M60-like family proteases. These proteins have a predicted signal peptide for periplasmic secretion and possess an M60 family catalytic domain, which contains a metal-binding site that accommodates Zn2+; this motif is characterized by a HEXXH peptide sequence and may have key mucolytic activity (Nakjang et al., 2012). As a metal ion-binding domain of the M60 protease family, HEXXH facilitates the hydrolysis of protein peptides through coordination with Zn2+ and imidazolyl groups on two histidine residues. Notably, these enzymes recognize not only specific peptide residues in protein substrates, but recent studies have revealed that their substrate specificity also depends on O-glycosylation modifications, a defining characteristic of mucinases (Nesta et al., 2014). As demonstrated by our mass spectrometry results, all three M60 enzymes target mucin-domain glycoproteins but produce distinct digestion banding patterns. However, the molecular mechanisms governing protease activity relative to both peptide sequence and glycosylation status remain unresolved. Several principles partially explain the substrate specificity of these mucinases. All three enzymes contain carbohydrate-binding modules (CBMs), which are characteristic of the multi-CBM architecture of M60 mucinases. Crystallographic structure PDB:7BLH demonstrates that the C-terminal CBM domain, residues K225–K367 of BT3015, binds β-D-galacturonic acid glycans (Costa and Carvalho, 2021). This binding is critical for recognizing mucin O-glycosylation cores. This structural motif belongs to the F5/8-type C domain classification. Such domains exhibit high polysaccharide affinity and bind gastric mucins (Shinya et al., 2016; Lawrence et al., 2012; Ficko-Blean et al., 2012). However, these enzymes selectively degrade mucin-domain glycoproteins but not O-glycosylated Fetuin-A. We propose that analogous to ZmpB, interspaced CBMs position the catalytic domain near exposed mucin regions by matching glycosylation periodicity (Pluvinage et al., 2021). CBMs recognize and bind polysaccharide side chains. This carbohydrate-targeting hydrolase activity could enable the development of cancer-specific mucin therapies that are specific to glycosyl side chains (Dingjan et al., 2015).

Notably, cytological experiments of mucinase-treated mammalian cell lines have shown varying results depending on the cell line, but these preliminary data do not yet fully elucidate the comprehensive effects of these proteases on complex cellular systems, particularly the underlying mechanisms driving the observed inhibition of tumor cell growth. However, based on the literature and our preliminary observations, we hypothesize that these proteases may exert their cell type-specific effects by selectively cleaving glycoproteins with cell type-specific glycosylation patterns—particularly those that are overexpressed or aberrantly modified in tumor cells. Such cleavage events could disrupt critical cellular processes, including cell proliferation, adhesion, and interactions with the microenvironment, ultimately leading to the observed phenotypic abnormalities. We plan to investigate these potential mechanisms in depth in the future.

This study demonstrates the substrate specificity of M60 family mucinases in *B. theta* and supports the development of mucolytic agents. Owing to the diffuse distribution of PMP mucus in the peritoneal cavity, vital organs (e.g., liver and kidney) may be susceptible to off-target effects of mucolytic drugs. Comprehensive characterization of mucinase kinetics is therefore essential. Through molecular dynamics simulations of mucinase-digested glycoproteins, Chongsaritsinsuk’s research predicted conformational changes to elucidate functional mechanisms, advancing glycoprotein research (Chongsaritsinsuk et al., 2023). However, direct techniques for revealing these processes are lacking: (a) Mass spectrometry cannot simultaneously resolve peptide sequences and glycosylation modifications. (b) Efficient separation methods for biological macromolecules remain underdeveloped. These technical limitations significantly limit mucinase research and therapeutic development. The development of advanced analytical approaches is consequently imperative.

## Conclusion

Since the pathogenesis of PMP, a malignant mucinous cancer, remains unknown, research on its targeted therapy has been limited. Conventional clinical treatments still have significant room for improvement. Our approach begins with mucolysis. Using PMP mucus as a growth medium for the probiotic bacterium *B. theta*, we successfully isolated three highly active mucinases with specificity for O-glycosylated mucin domains. These enzymes, particularly BT3015, which liquefied and dissolved gel-like mucus without supplementary factors, demonstrated excellent *in vitro* mucolytic activity. Critically, these mucinases do not degrade non-glycoproteins or non-mucinous proteins and exhibit favorable targeting properties. This study supports the potential of probiotic-derived products for developing mucolytic drugs for malignant mucinous cancers, especially PMP. Subsequent research will further explore these applications and investigate the directed evolution of mucinases to enhance degradation activity and broaden substrate specificity. This approach may expand novel options for targeted cancer therapy.

## Data Availability

The transcriptome raw data generated in this study have been submitted to the NGDC database (https://ngdc.cncb.ac.cn/gsa), project number PRJCA047578. The mass spectrometry data is available in the MassIVE database (https://massive.ucsd.edu/), accession MSV000101498.
