# Supplementary material for: Mucinase from Bacteroidesthetaiotaomicron as mucolytic for mucinous cancer pseudomyxoma peritonei
Source: Front Microbiol. 2026 May 1;17:1722421. doi: 10.3389/fmicb.2026.1722421 (PMC13176161; doi:10.3389/fmicb.2026.1722421)
Supplement: Supplementary file 1 [file Supplementary_file_1.docx]

supplementary document-1

B.theta strains and culture conditions

Commercially purchased B. theta (ATCC 29148) lyophilized strain (Beina Biotechnology, Beijing, CN) was grown statically at 37°C on 90mm Columbia blood agar plate(HUANKAI #CP0160) which in a anaerobic chamber jar with AnaeroPack (MGC Company, Tokyo, JP). The colonies were scraped off the plates and suspended in PBS. After centrifugation at 5000rpm, the B. theta precipitate was cleaned again with PBS. About 1*10^8 cfu/ml cells were suspension in FT medium (15.0g/L peptone, 5.0g/L yeast extract powder, 5.0g/L glucose, 0.5g/L sodium thioglycolate, 0.5g/L L-cystine, 2.5g/L sodium chloride, 0.001g/L resazurin, 0.75g/L agar, pH 7.1±0.2 under 25℃) containing 20% glycerol, store at -80℃.

Acquisition and purification of PMP mucus samples:

Anonymized PMP mucus samples were donated under the informed consent of the patients, and were available for medical research. The mucus samples were initially cleaned, subjected to stepwise salt precipitation to remove free impurities, and dialyzed with ultrapure water (Milli-Q, Millipore) to re-gel and lyophilize (see appendix for details). The lyophilized samples were sterilized by ^60^Co source irradiation (Huayuan Nuclear Radiation Technology Co., Ltd., Yunnan, CN). The samples were mixed and ground in liquid nitrogen, weighed and packaged in a sterile cleaning bench, samples were stored at -80°C before use.

Isolate RNA, prepare and sequence RNA-Seq cDNA libraries

Refer to the growth curves that have been measured, logarithmic growth phase (27h) bacterial cultures were harvested for RNA-seq. If necessary, we also conducted interval sampling at different time points, but apart from the differences in sampling times, all experimental methods related to RNA extraction remained consistent.

For each cultures, transfer medium to sterilized 50ml centrifuge tubes, supplemented with pre-cooled PBS, and centrifuged at 8000rpm for 20min in 4℃. Add 10ml protease K solution (0.2mg/ml) to re-suspend precipitation and incubate for 5 minutes. Collect the precipitation after centrifuge again, add 2ml RNA-Wait (Biosharp LIFE SCIENCES #BL621A), and pace on ice for 10min. The final samples were stored at -80°C until RNA extraction.

Transport frozen samples on dry ice for RNA-seq by CSC Genomics Ltd (Shanghai, CN). Incubated with 2mg/ml lysozyme for 5 min after centrifuged, cells were extracted for library construction and sequencing. Isolate total RNA from all samples using the RNeasy Mini Kit (Qiagen #74106) according to the manufacturer's instructions; Determine RNA integrity numbers using the 2100 Bioanalyzer System (Agilent Technologies, CA, USA) to assess sample quality, and using Qubit® 3.0 (Life Technologies, CA, USA) and Nanodrop RNA-seq strand-specific libraries were constructed using the VAHTS Total RNA-seq (H/M/R) Library Prep Kit (Vazyme, China) according to the manufacturer’s instructions. Briefly, RNA was purified by magnetic beads after removal of rRNA. Then RNA was fragmented into small pieces using divalent cations for 8 min at 94℃. The cleaved RNA fragments were copied into first strand cDNA using reverse transcriptase and random primers. Second strand cDNA synthesis was subsequently performed using DNA Polymerase I and RNase H. These cDNA fragments then go through the end repair process, addition of a single ‘A’ base, and ligation of the adapters. The products were purified and enriched by PCR to create the final cDNA library. Purified libraries were quantified and validated by Qubit® 3.0 Fluorometer and Agilent 2100 bioanalyzer to confirm the insert size and calculate the mole concentration. Cluster was generated by cBot after the library diluted to ten pM and then were sequenced on the Illumina NovaSeq 6000 platform (Illumina, USA).

Identification of differentially expressed genes (DEGs) and database enrichment analysis

Raw reads from the resulting download data were filtered and cleaned and compared to the B. theta reference genome (GCF_014131755.1_ ASM1413175v1 _genomic.fna) using Bowtie2. Gene abundance was expressed as fragments per kilobase of exon per million reads mapped (FPKM). Stringtie software was used to count the fragment within each gene, and TMM algorithm was used for normalization. Differential expression analysis for mRNA was performed using R package edgeR. Differentially expressed RNA with |log2(FC)| value >1.5 or >2 and q value <0.05, considered as significantly modulated, were retained for further analysis. This choice is motivated by the decision to maximize the sensitivity of this analysis, to perform a massive screening and identify candidate genes to be validated with a wider sample population with real-time PCR analysis.

qRT-PCR verify gene of M60 family mucinase and PULs associated with Mucus degradation

The B.theta were centrifuged and collected using the same methods as those employed in the sequencing pipeline. Subsequently, RNA was extracted and reverse transcribed into cDNA utilizing Trizol (Invitrogen #15596026) and the PrimeScript™ RT reagent Kit (Takara #RR037A). The resulting cDNA was then purified using magnetic beads, with its concentration assessed by Qubit® 3.0 and purity determined via nanodrop 2000 microspectrophotometer analysis. Gene expression levels were evaluated through qRT-PCR, normalized against 16S rDNA. The reaction was performed using the TB Green® Premix Ex Taq™ kit (Takara #RR420Q), following manufacturer's instructions, and quantified and analyzed in a Quantstudio ®7 pro (Thermo Fisher) real-time fluorescence instrument. Primers required for qRT-PCR experiments are listed in supplementary document table 1.

Mucinase structure compare

The structural relativity of BT3015 and BT4272 to the known BT4244 is find out by the sequence homology of the enzyme activity domain. MEGA11 software was applied to search for the similarity of enzyme activity domain sequences of proteins through comparison based on Blast algorithm.

In addition to the motif-based homology comparison, the Pairwise Structure Alignment based on RCSB proposed by Sebastian Bittrich to compare protein 3D structures. The analysis was obtained by manual search, using the existing predicted Bacteroides thetaiotaomicron protein AlphaFold model from the UNIPROT database, and traversing the proteome-wide search.

The final highest comparison score proteins was used to search and establish motif with Discovered Motifs feature in MEME suite, identify common motifs consisting of 4-50 amino acids. The results unveiled the top-10 motifs with the highest level of confidence in different color, that guide the visualization and highlighted for active domains of protein structure in Pymol. The numbers at the end of the tree show the evolutionary distances obtained by comparing amino acid sequences between different above-mentioned proteins by MEGA11.

M60 mucinase recombinant vector construction and protein purification:

Three M60 mucinase proteins were cloned from bacteria using a long primer pair containing the homology arm of the pBAD-HIS A plasmid, and expression vectors were constructed by seamless cloning. One copy of the 8*His-tag was introduced into the N-terminal end of the ORF through the homology arm, and the original 6*His-tag in the vector was not included , the signal peptide of the target protein predicted by SignaI5.0 was removed in the vector construction. Primers are listed in supplementary document table 2.

The correctly sequenced plasmid was transformed into E. coli Top10 competence cell for expression. That is, the strain containing the target gene was induced overnight at 37°C in LB basic medium and then inoculated into 1000ml culture flasks at 1:100 and grown in a 200-rpm shaking incubator at 37°C only with OD600 reaching 0.6-0.8. Add L-arabinose to a final concentration of 10mg/L to induce the expression of BT3015, BT4244 and BT4272.

Cell precipitates were collected after 3h of sustained induced expression and lysed in lysis buffer containing 50 mM NaH_2_PO_4_, 100 mM NaCl, 10 mM imidazole, 15% Glycerol, bound by manual loading into a Ni-NTA resin, and gradient eluted by a addition of 250 mM imidazole in lysis buffer. The eluted target proteins were purified again with Superdex-200 in SEC method. Store these target proteins at -80°C in PBS which contain 20% glycerol.

Morphological observation of mammalian cells treated with M60 mucinase.

The process of treating mammalian cultures with drugs was consistent with the methods described in the main text, but unlike the cell viability assay, the cell morphology was observed using an IncuCyte S live-cell imaging system with a 10X objective，and are 100 cells per well at the beginning to incubate. Typical results from each cell treatment are shown in Supplementary Fig 7.

supplementary document table 1：

Table 1. primers used in qRT-PCR for Puls and genes expression identification:

| Sequence Definition | Sense Primer | Anti-sense Primer |
| --- | --- | --- |
| BT4240 | AGACCTGAATCGTGTGAACT | TTGATATAATCGGCAAGGAACC |
| BT4241 | GGATGGAACCGAATACTTCAA | TTGTCTTCTGTGCTTACCTTC |
| BT4242 | GAGGAGATAGGACGACACAT | TCGGAAGATGATGGTAGACTC |
| BT4243 | TCAGCAAGATGTAATCCAACTC | ATATGTGTCCAGCGTTCAAC |
| BT4244 | CCATCACAAGTCAACAACCA | CGCCGCCATTACATTATCTT |
| BT4245 | GCGACAATTATGACGATACCT | ATAGTTAGTAGCCTCTCCTGTT |
| BT4246 | GCTTATTACCACTTCGCTACTT | GCAATCCAGTCTACACAATAGT |
| BT4247 | TTATAGACGCAGTGGACGATA | CGACTACGACAACTTCATTCA |
| BT4248 | ATTGGAAGGACGGAAGATACA | GATGCTGCCTGTGAATGAAG |
| BT4249 | ATTAAAGGTTTGCTGGCAGG | AGTTCTCTATCCACTGATTCCA |
| BT4250 | GGAATTGGATGCTGCTGATT | GCTAATGTTATCTGCCGTTCT |
| BT4272 | ACATCAAGCCTCAAGGAAGT | CCATCTCGTCGCAACTGA |
| BT3015 | CGGTGACGGACAAGGATT | ACTCGCTGATACGGAACTC |
| 16S(515F+806R) | GTGYCAGCMGCCGCGGTAA | GGACTACNVGGGTWTCTAAT |

supplementary document table 2：

Table 2. primers used in M60 family proteins expression vector construction :

| Sequence Definition | 5’——>3’ sense |
| --- | --- |
| pBAD-F | tgagttcaaacggtctccagc |
| pBAD-R | GTGGTGATGATGGTGATGGTGGTGcatggttaattcctcctgttagcc |
| pBT4244-F | CACCACCATCACCATCATCACCACtgcaaggataccgaaaaatc |
| pBT4244-R | gctggagaccgtttgaactcattataacagaatacgttttccgt |
| pBT3015-F | CACCACCATCACCATCATCACCACtgctctgacgatgacggctc |
| pBT3015-R | Gctggagaccgtttgaactcatcaattcattggaatgtcgatacgtt |
| pBT4272-F | CACCACCATCACCATCATCACCACtgcaagagtgatgataaggag |
| pBT4272-R | gctggagaccgtttgaactcactatttatttgctttaatacgttgc |


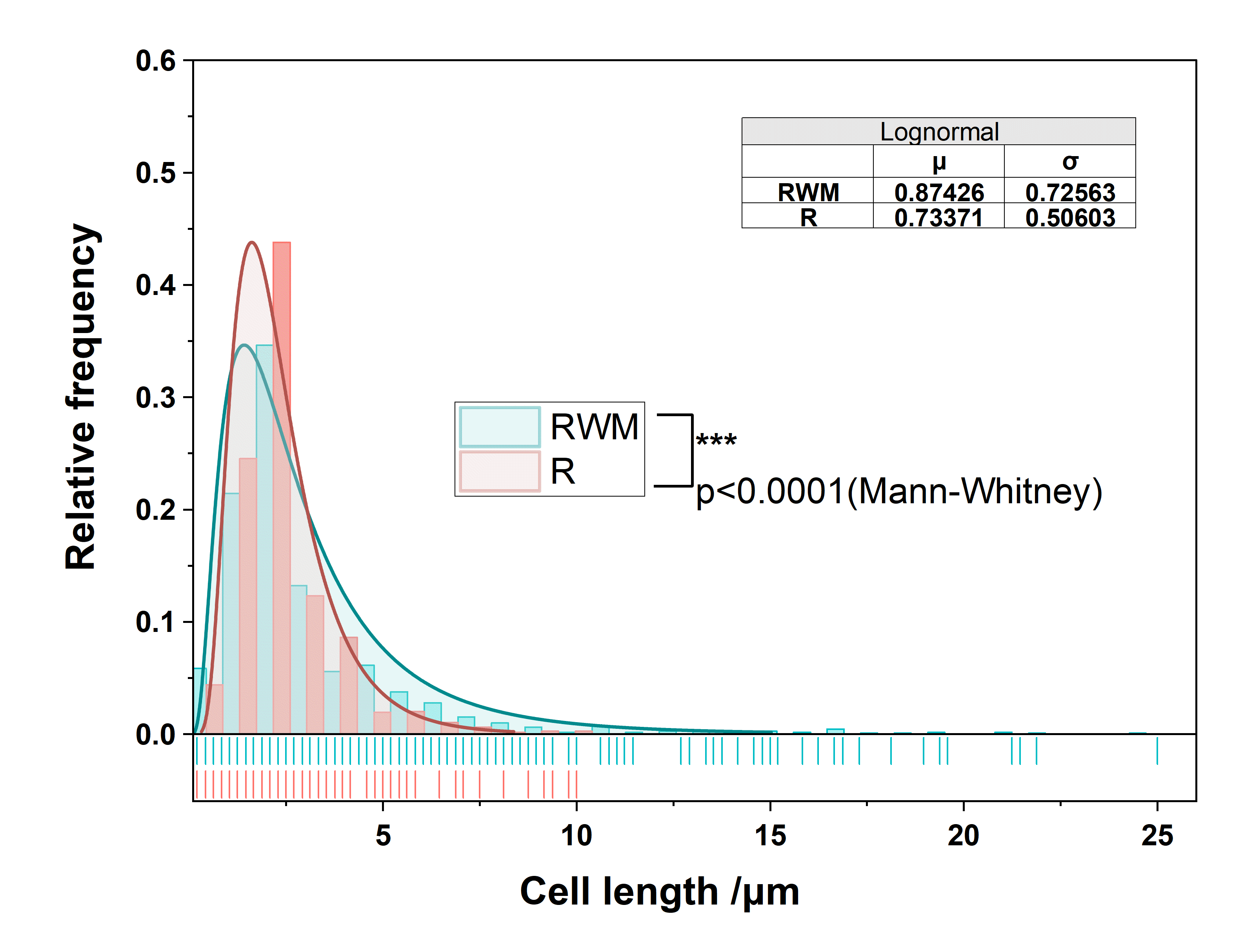


**Sup Fig 1: Distribution frequency of B.theta cell length in R/RWM group.**

The B.theta bodies were significantly differentiated between the experimental group (RWM) and the control group (R). The length of B.theta cultured with mucus supplementation can reach longer. B.theta in theRWM which in 5-10um zone accounted in a higher proportion. The optical microscopy images of each group of bacterial smears stained with methyl blue were analyzed using imageJ, the frequency distribution map was drawn after the distribution of B.theta length was counted in originLab. The results in both groups were fitted using log-normal distributions. Analysis of variance was performed using Mann-Whitney for raw data.


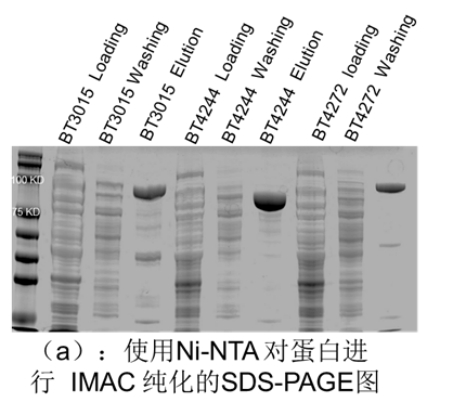


**Sup Fig 2: SDS-PAGE shows target proteins obtained from E. coli lysate by IMAC-resin Ni-NTA metal affinity.**

Loading in lanes 2, 5, and 8 represents the total protein from E. coli lysate. lanes 3, 6, and 9 represent the wash solution after combining the sample with wash buffer. elution in lanes 4, 7, and 10 indicates the use of a fixed concentration of eluent to eluate the target protein solution bound to the Ni-NTA resin.

| 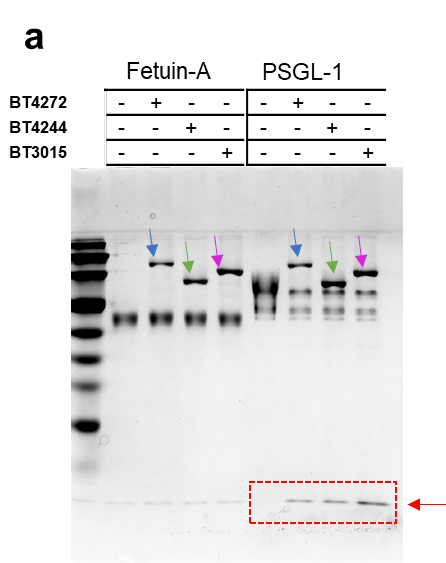 | 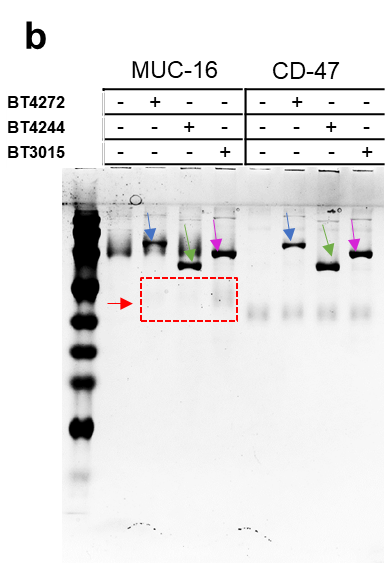 |
| --- | --- |
| 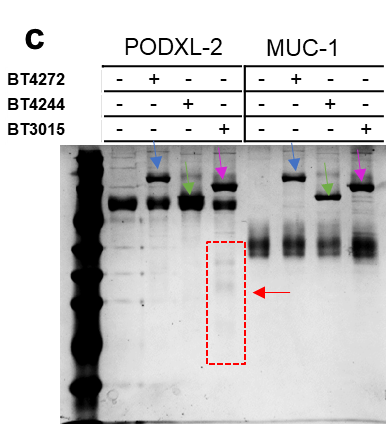 | 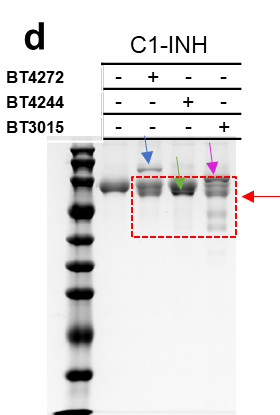 |
| 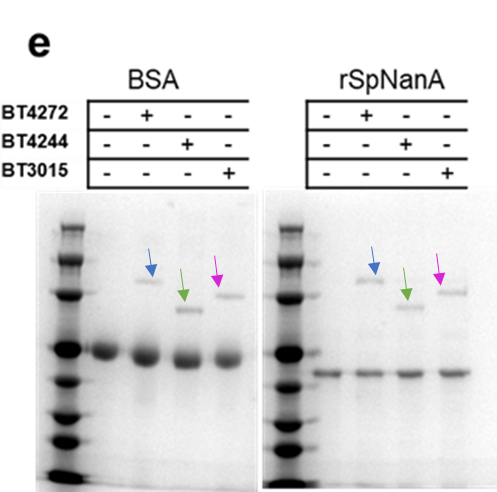 | |

**Sup Fig 3:** **The co-incubation of BT3015, BT4244 or BT4272 with different glycosylated substrate proteins showed the differential degradation of the corresponding substrates by the three proteins.**

Coomassie-stained SDS-PAGE electrophoresis gels showed differences in substrate degradation for BT3015, BT4244, and BT4272 when each substrate was incubated with different proteins alone or separately. The red box indicated by the red arrow indicates the degradation product band of the substrate protein. And the arrow which colored by blue, green or purple is pointed BT4272, BT4244 or BT3015 separately. The results are shown in groups of four bands with **a:** Fetuin-A and PSGL-1, **b:** MUC-16 and CD-47, **c:** PODXL-2 and MUC-1, **d:** C1-INH and **e:** Non-glycosylated proteins BSA and prokaryotic cell protein rSpNanA as substrates.

| 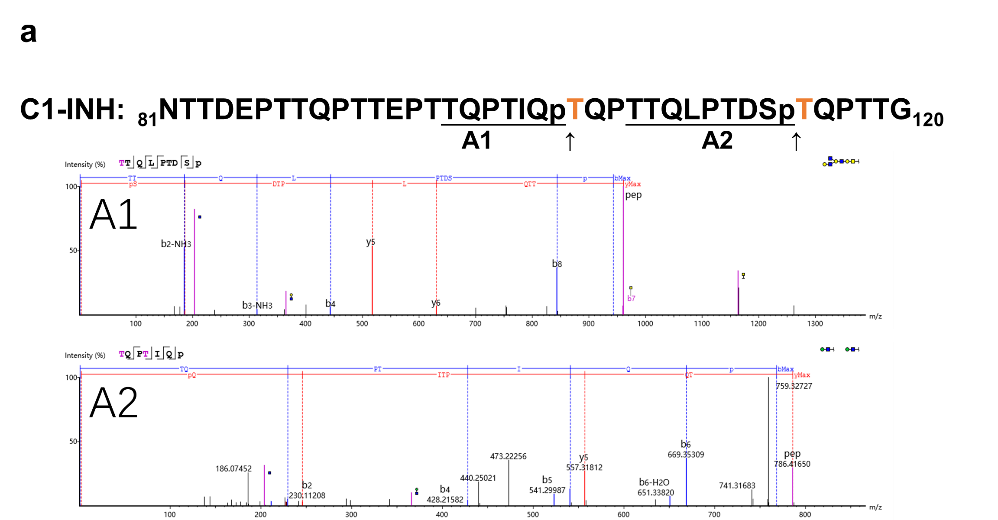 |
| --- |
| 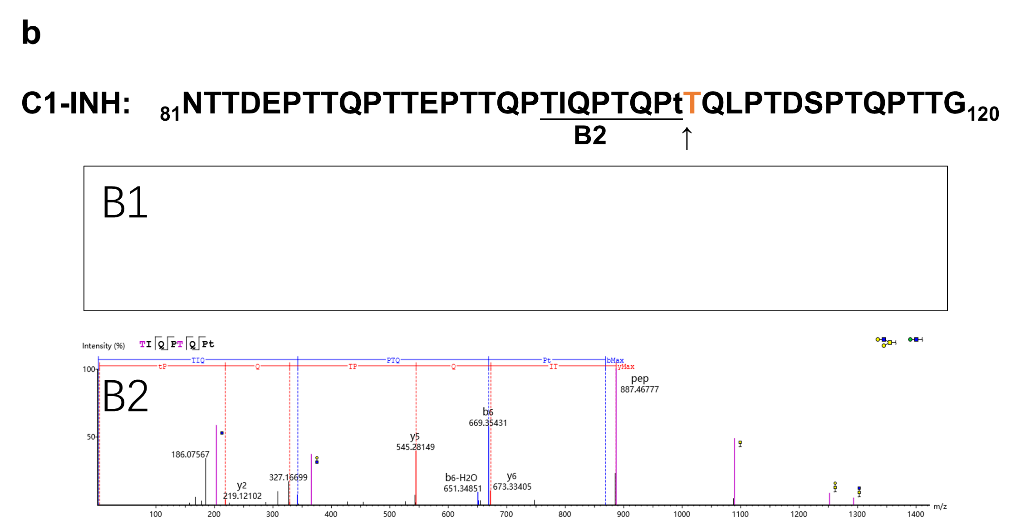 |
| 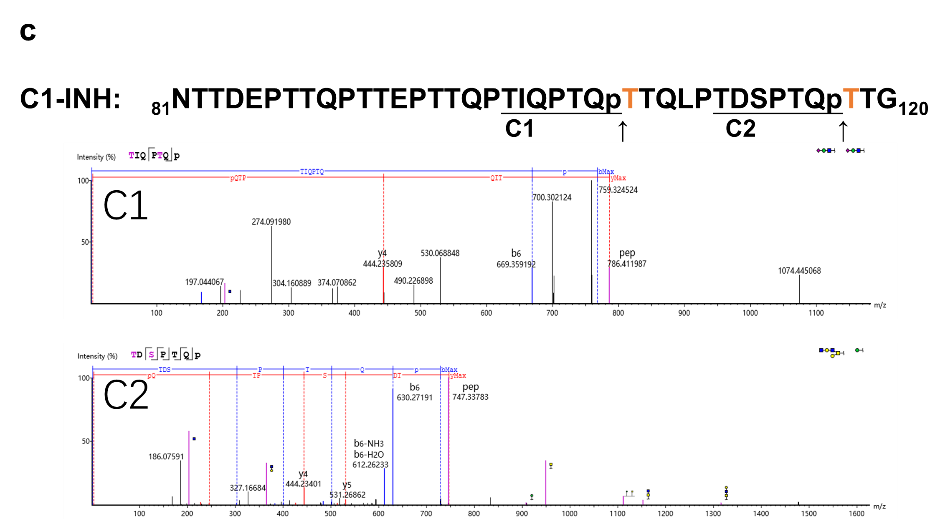 |
| **Sup Fig 4 Representative peptides and their MS2 mass spectra were identified at the cleavage sites of the m60 proteases BT3015, BT4244, and BT4272.**  Peptides of C1-INH treated with **a:** BT3015, **b:** BT4244, and **c:** BT4272 respectively, were analyzed using mass spectrometry to obtain characteristic cleavage sites for the three proteases treated with sialidase NA (A2, B2, and C2) or without sialidase NA treatment (A1, B1, and C1). The results indicate that the primary cleavage sites of the enzyme are located between the amino acid positions 81-120 of C1-INH, an area rich in P/T/S, which serves as a glycosylation site for C1-INH. The line below the sequence indicates a single peptide, and the corresponding mass spectrometry peak source for the peptide is shown with serial numbers, while arrows indicate the corresponding enzyme cleavage sites. In the treatment group BT4244 without added sialic acid (B1), no confident fragments could be obtained. Lowercase letters denote that the corresponding amino acid in the peptide segment is modified by ^18^O, and during enzyme-mediated protein cleavage, it always occurs at the N-terminus threonine which orange colored or serine (data not shown). |


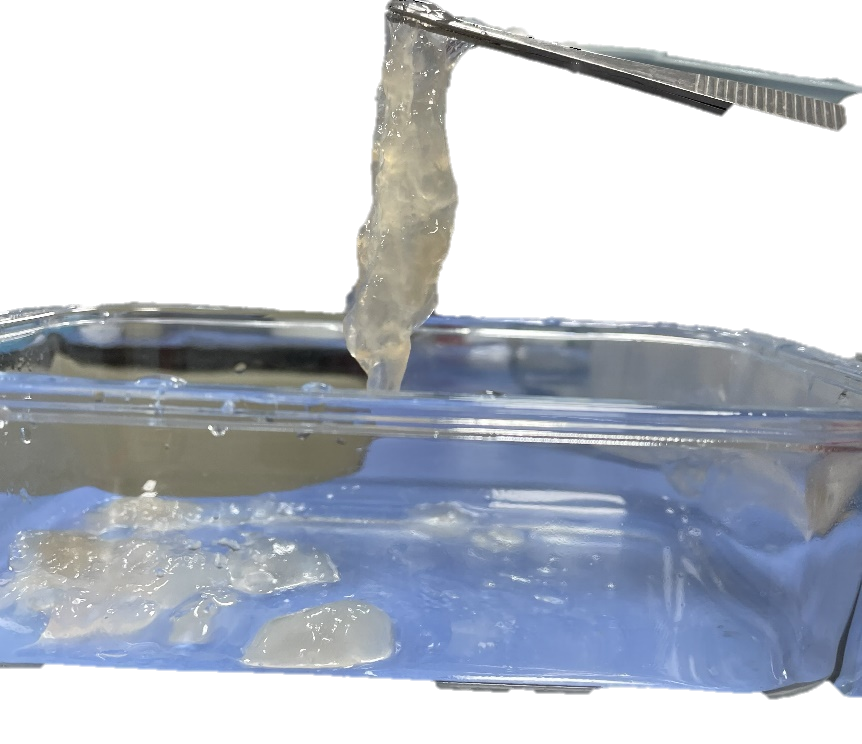


**Sup Fig 5 Mucus from patients with pseudomyxoma peritonei.**

The patient-derived mucus has a gel-like consistency, appearing as a colorless to amber-colored transparent semi-solid. It has high viscosity and is resistant to typical proteolytic hydrolysis.

**Sup fig 6 Expression characters in PULs-78 which BT4244 located and BT4272 as comparison.**


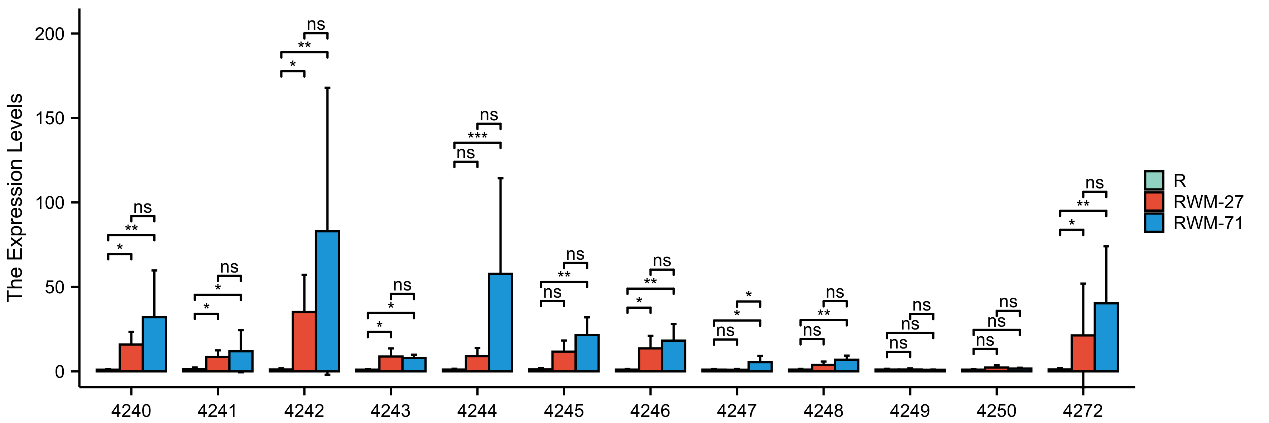


RNA transcription analysis of cultures with extended incubation times using the qRT-PCR method. Each data point represents the mean of three biological sample replicates and two technical replicates for each sample. Error: +SEM; *: p<0.05; **: p<0.01; ***: p<0.001. The different treatments of R and RWM represent whether PMP mucus is supplemented, while the number after RWM indicates the early (27h) and later (71h) cultures stage, respectively.

**Sup fig 7 Morphology of mammalian cells after M60 mucinase treatment.**


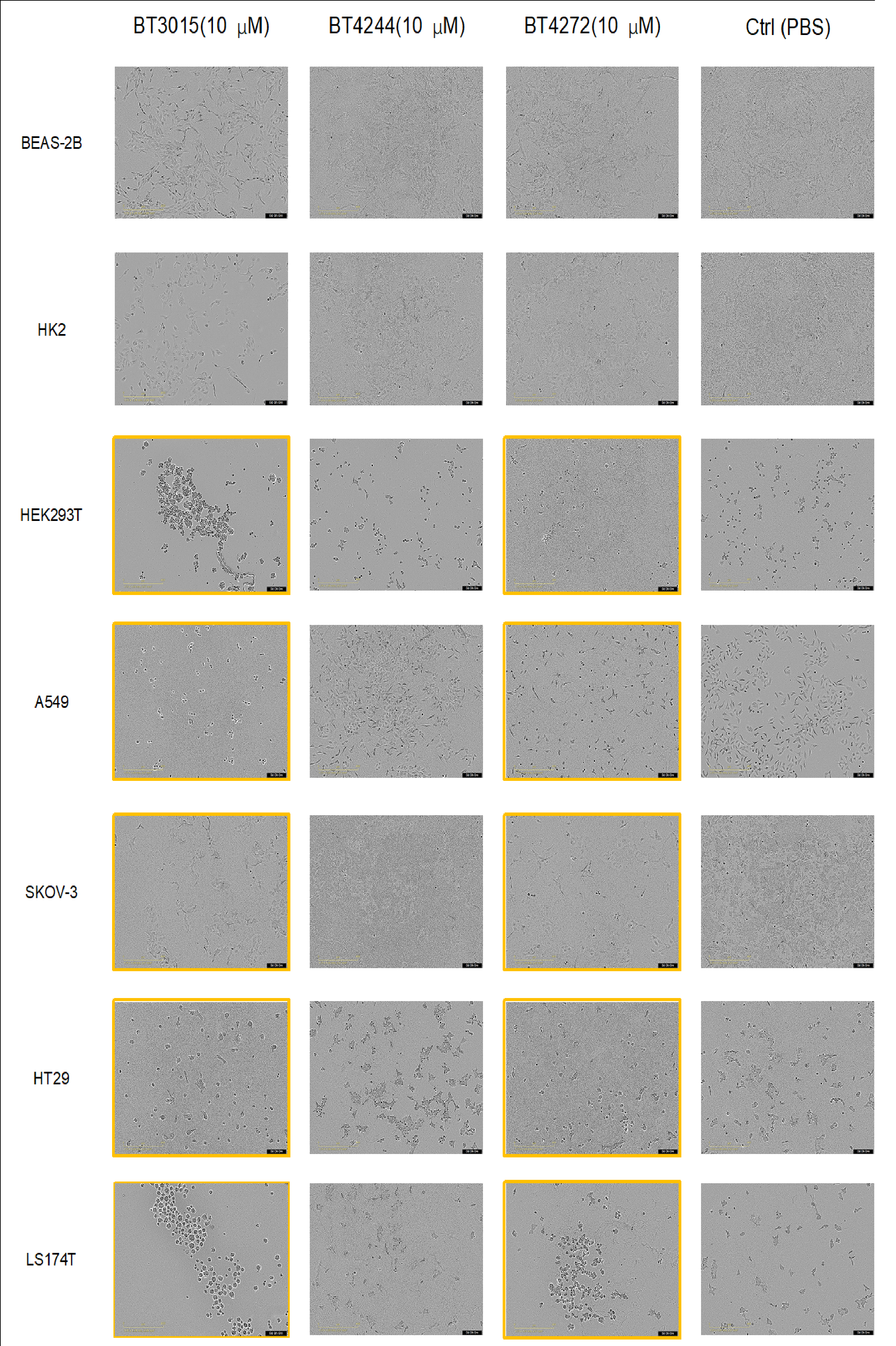
Microscopic images of cells that underwent significant morphological changes after mucinase treatment are marked with yellow boxes, including HEK293T, A549, LS174T, SKOV-3, and HT29.In several images, the camera focus is not accurate, which may cause the field of view to appear blurry, but cell counting and morphological analysis can still be performed, and it does not affect the final analysis results. This result shows cytotoxicity associated with cell type. However, there was no cytotoxicity was observed for the HK2 and BEAS-2B cells used in this experiment (High-resolution images will be provided in the original file format.).
