# Supplementary material for: Mucinase from Bacteroidesthetaiotaomicron as mucolytic for mucinous cancer pseudomyxoma peritonei
Source: Front Microbiol. 2026 May 1;17:1722421. doi: 10.3389/fmicb.2026.1722421 (PMC13176161; doi:10.3389/fmicb.2026.1722421)
Supplement: Supplementary file 2 [file Supplementary_file_2.docx]

## **Original Uncut Gel Images**

1. figure 4c：





* This image in the main-text is flipped horizontally to facilitate image annotation.

**Figure 4c attached in main text：**


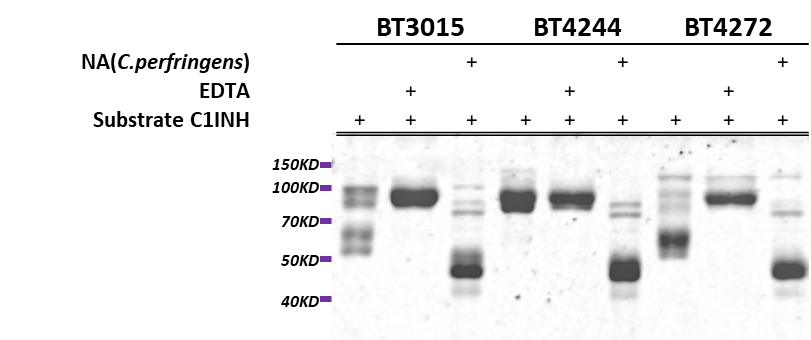


1. figure 4d：


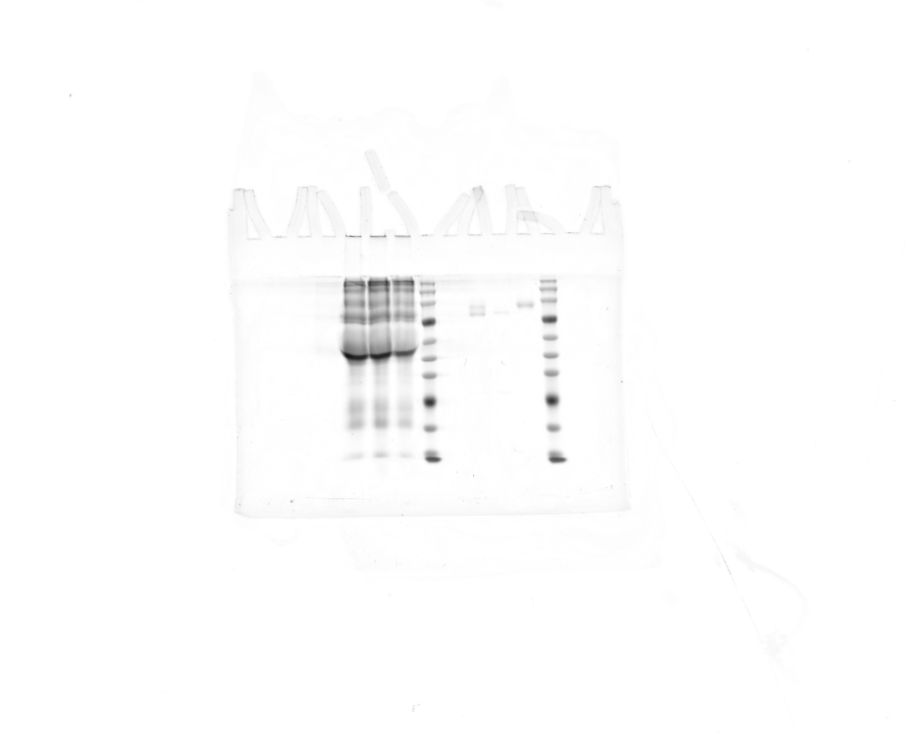


* This image in the main-text is flipped horizontally to facilitate image annotation. And the four lanes on the left containing markers are unrelated to this study, and cut out of in the main-text figure.

**Figure 4d attached in main text：**


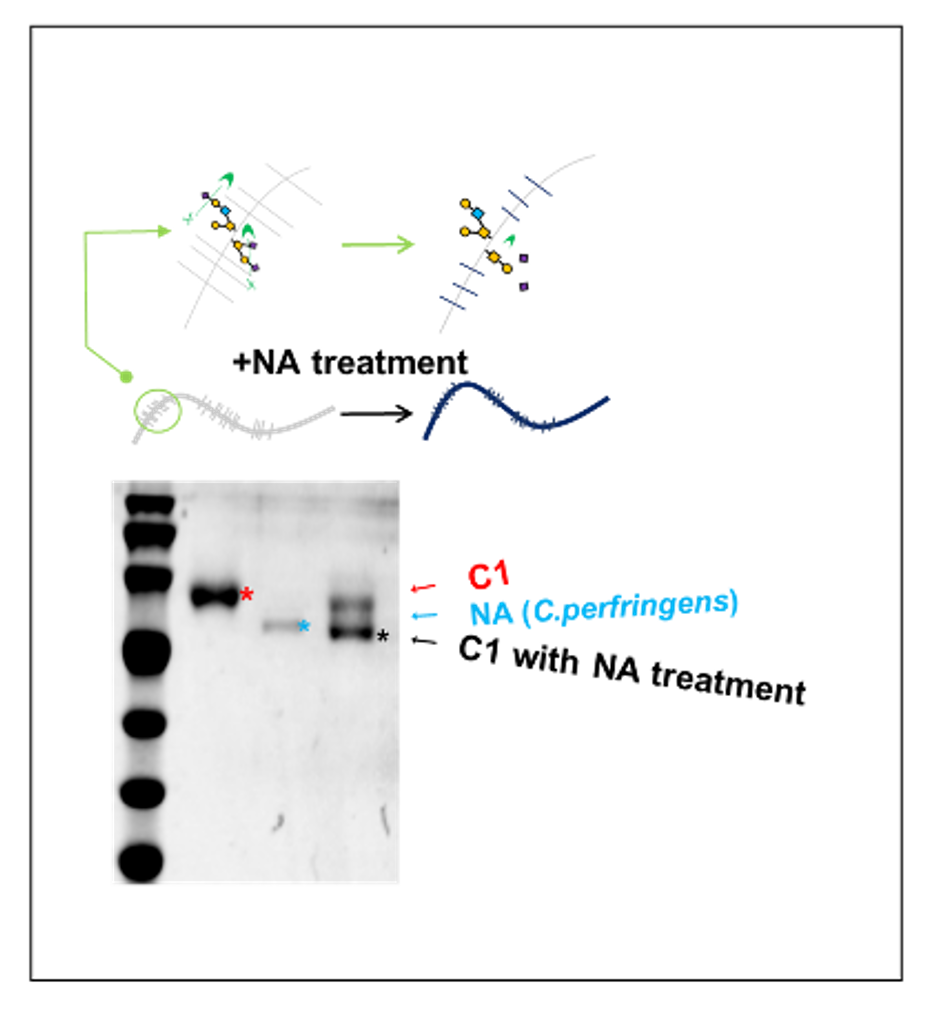


1. Sup Fig 2：


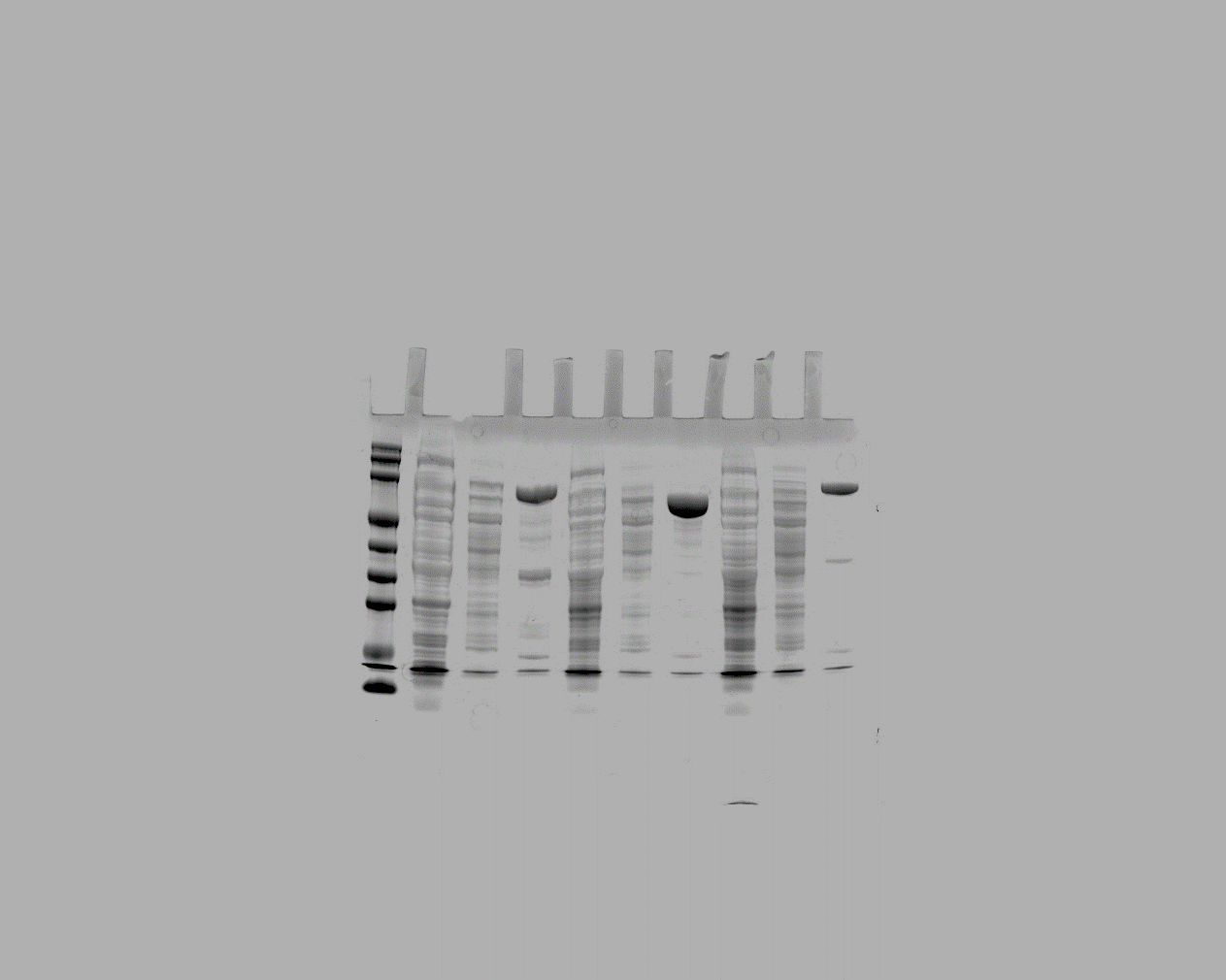


**Sup Fig 2 attached in supplementary Information document ：**


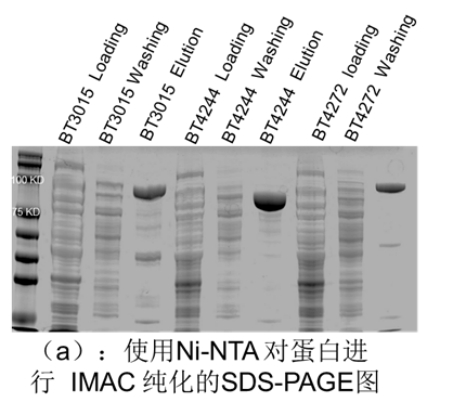


**Sup Fig 2: SDS-PAGE shows target proteins obtained from E. coli lysate by IMAC-resin Ni-NTA metal affinity.**

Loading in lanes 2, 5, and 8 represents the total protein from E. coli lysate. lanes 3, 6, and 9 represent the wash solution after combining the sample with wash buffer. elution in lanes 4, 7, and 10 indicates the use of a fixed concentration of eluent to eluate the target protein solution bound to the Ni-NTA resin.

1. Sup Fig 3: a,b,c,d


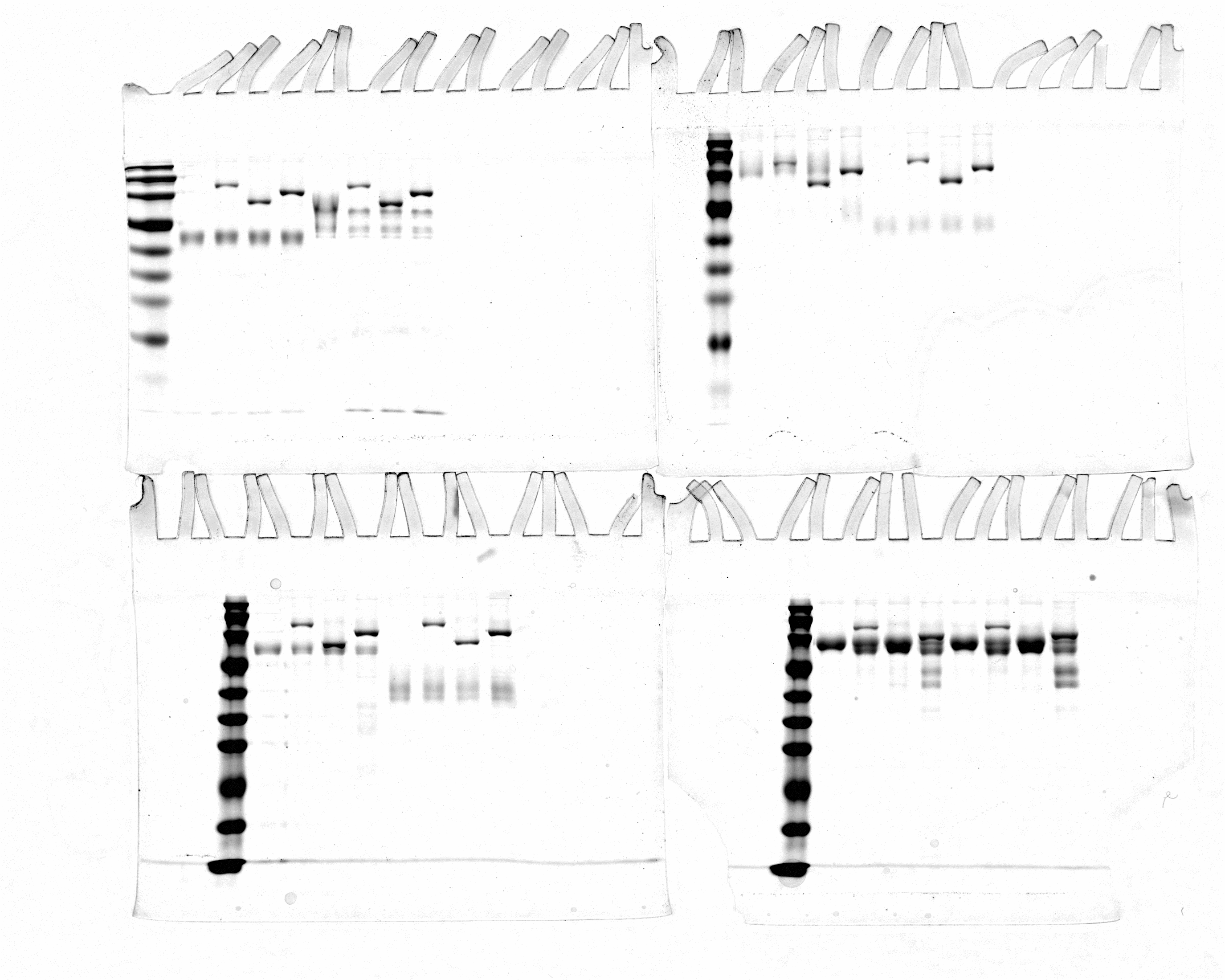


* Sup Fig 3d which in supplementary document only shows one of twice repeat experiment

1. ：Sup Fig 3:e


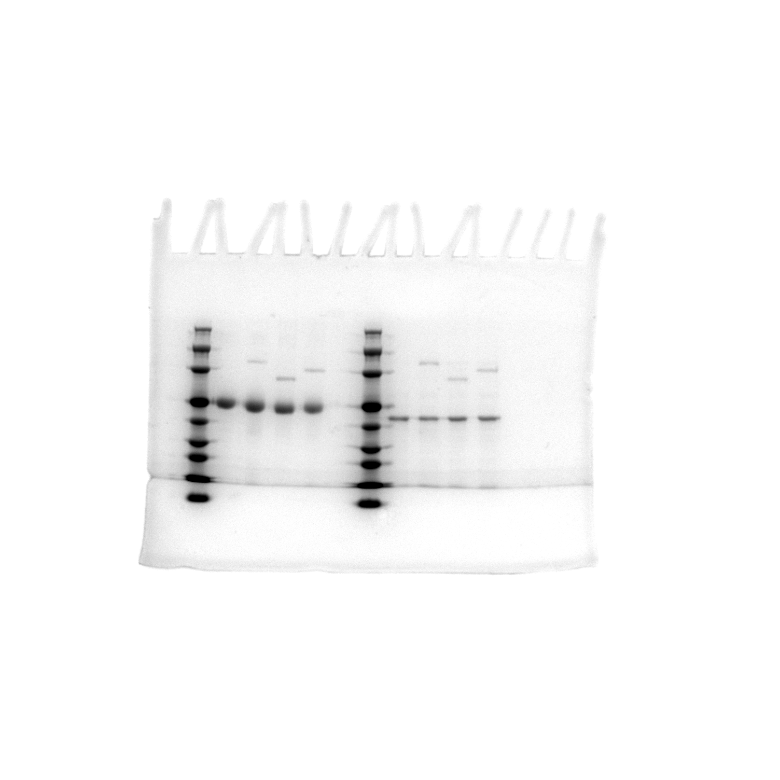


**Sup Fig 3（a-e）attached in supplementary Information document ：**

| 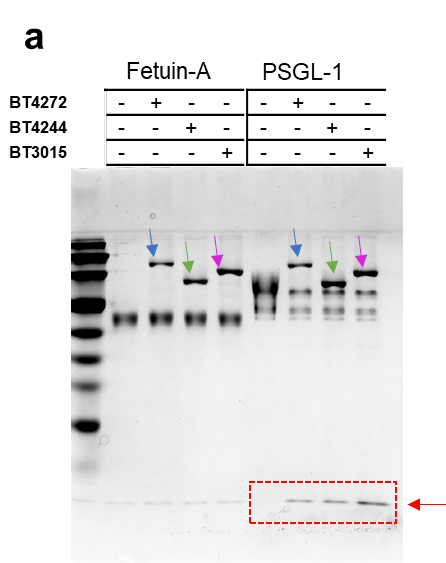 | 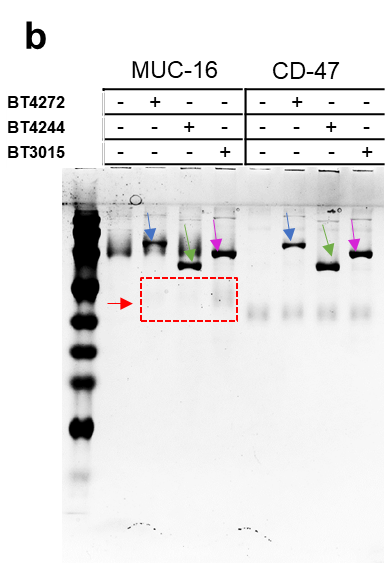 |
| --- | --- |
| 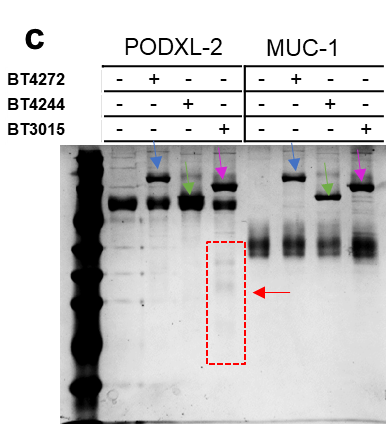 | 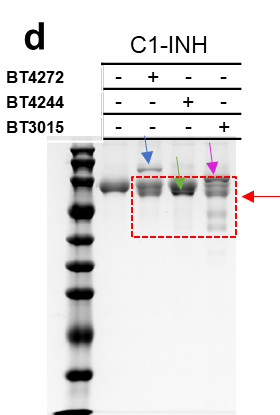 |
| 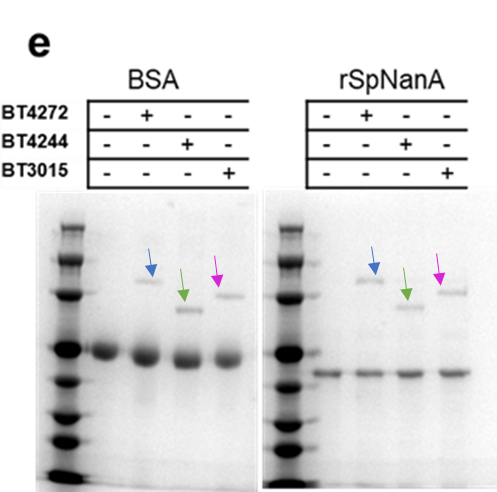 | |

**Sup Fig 3:** **The co-incubation of BT3015, BT4244 or BT4272 with different glycosylated substrate proteins showed the differential degradation of the corresponding substrates by the three proteins.**

Coomassie-stained SDS-PAGE electrophoresis gels showed differences in substrate degradation for BT3015, BT4244, and BT4272 when each substrate was incubated with different proteins alone or separately. The red box indicated by the arrow indicates the degradation product band of the substrate protein. The results are shown in groups of four bands with **a:** Fetuin-A and PSGL-1, **b:** MUC-16 and CD-47, **c:** PODXL-2 and MUC-1, **d:** C1-INH and **e:** Non-glycosylated proteins BSA and prokaryotic cell protein rSpNanA as substrates.
